# Periostin promotes ovarian cancer metastasis by enhancing M2 macrophages and cancer-associated fibroblasts via integrin-mediated NF-κB and TGF-β2 signaling

**DOI:** 10.1186/s12929-022-00888-x

**Published:** 2022-12-22

**Authors:** Sheng-Chieh Lin, Yi-Chu Liao, Po-Ming Chen, Ya-Yu Yang, Yi-Hsiang Wang, Shiao-Lin Tung, Chi-Mu Chuang, Yu-Wen Sung, Te-Hsuan Jang, Shuang-En Chuang, Lu-Hai Wang

**Affiliations:** 1 Graduate Institute of Integrated Medicine and Chinese Medicine Research Center, China Medical University, No. 91, Hsueh-Shih Road, Taichung, 40402 Taiwan; 2 Graduate Institute of Biomedical Sciences, China Medical University, Taichung, Taiwan; 3 Institute of Molecular and Genomic Medicine, National Health Research Institutes, Miaoli, Taiwan; 4 Research Assistant Center, Show Chwan Memorial Hospital, Changhua, Taiwan; 5 National Institute of Cancer Research, National Health Research Institutes, Miaoli, Taiwan; 6 Institute of Molecular Medicine, National Tsing Hua University, Hsinchu, Taiwan; 7 Department of Hematology and Oncology, Ton-Yen General Hospital, Hsinchu, Taiwan; 8 Department of Nursing, Hsin Sheng Junior College of Medical Care and Management, Taoyuan, Taiwan; 9 Department of Obstetrics and Gynecology, Taipei Veterans General Hospital, Taipei, Taiwan; 10 School of Medicine, National Yang-Ming University, Taipei, Taiwan; 11 Department of Obstetrics and Gynecology, China Medical University Hospital, Taichung, Taiwan

**Keywords:** Ovarian cancer, Metastasis, Periostin, Macrophages, Cancer-associated fibroblast, NF-κB, TGF-β2

## Abstract

**Background:**

Ovarian cancer has the highest mortality among gynecological cancers due to late diagnosis and lack of effective targeted therapy. Although the study of interplay between cancer cells with their microenvironment is emerging, how ovarian cancer triggers signaling that coordinates with immune cells to promote metastasis is still elusive.

**Methods:**

Microarray and bioinformatics analysis of low and highly invasive ovarian cancer cell lines were used to reveal periostin (POSTN), a matrix protein with multifunctions in cancer, with elevated expression in the highly invasive cells. Anchorage independent assay, Western blot, RNA interference, confocal analysis and neutralizing antibody treatment were performed to analyze the effects of POSTN on tumor promotion and to explore the underlying mechanism. Chemotaxis, flow cytometry and cytokine array analyses were undertaken to analyze the involvement of POSTN in cancer-associated fibroblast (CAF) and macrophage modulation. Correlations between POSTN expression levels and clinical characteristics were analyzed using the Oncomine, commercial ovarian cancer cDNA and China Medical University Hospital patient cohort. In vivo effect of POSTN on metastasis was studied using a mouse xenograft model.

**Results:**

Expression of POSTN was found to be elevated in highly invasive ovarian cancer cells. We observed that POSTN was co-localized with integrin β3 and integrin β5, which was important for POSTN-mediated activation of ERK and NF-κB. Ectopic expression of POSTN enhanced whereas knockdown of POSTN decreased cancer cell migration and invasion in vitro, as well as tumor growth and metastasis in vivo. POSTN enhanced integrin/ERK/NF-κB signaling through an autocrine effect on cancer cells to produce macrophage attracting and mobilizing cytokines including MIP-1β, MCP-1, TNFα and RANTES resulting in increased chemotaxis of THP-1 monocytes and their polarization to M2 macrophages in vitro. In agreement, tumors derived from POSTN-overexpressing SKOV3 harbored more tumor-associated macrophages than the control tumors. POSTN induced TGF-β2 expression from ovarian cancer cells to promote activation of adipose-derived stromal cells to become CAF-like cells expressing alpha smooth muscle actin and fibroblast activation protein alpha. Consistently, increased CAFs were observed in POSTN overexpressing SKOV3 cells-derived metastatic tumors. In clinical relevance, we found that expression of POSTN was positively correlated with advanced-stage diseases and poor overall survival of patients.

**Conclusions:**

Our study revealed a POSTN-integrin-NF-κB-mediated signaling and its involvement in enhancing M2 macrophages and CAFs, which could potentially participate in promoting tumor growth. Our results suggest that POSTN could be a useful prognosis marker and potential therapeutic target.

**Supplementary Information:**

The online version contains supplementary material available at 10.1186/s12929-022-00888-x.

## Background

According to World Health Organization (WHO) Classification of Tumors, ovarian cancer has been categorized into (1) surface epithelial-stromal tumor represents over 80% of all ovarian cancer, (2) sex cord-stromal tumor, (3) germ cell tumor and (4) mixed tumors. Epithelial ovarian cancer (EOC) has the highest mortality among gynecological malignancies since about 70% of ovarian cancer patients are diagnosed with advanced-stage disease having already distant metastasis and with 5-years survival rate about 27% [1]. The EOC can be subdivided according to histology and origin of cells into 4 major types, namely, serous (70–80%), mucinous (10%), endometrioid (5%) and clear cell (3%) tumors. The histopathology of ovarian cancers is heterogeneous and complex; besides, lack of specific biomarker further makes it difficult for targeted therapy [2]. Current standard treatment for advanced ovarian cancer undertakes cytoreduction surgery, which removes the primary tumor and visible intraperitoneal nodules, followed by chemotherapy such as platinum compounds (cisplatin or carboplatin) and microtubule inhibitor paclitaxel. Although initial response to the chemotherapy is in general satisfactory, resistance and recurrence, especially metastatic recurrence, inevitably appears in most cases accounting for most of the mortality. Thus, better understanding of the basis for ovarian cancer metastasis, as well as identification of useful biomarkers and development of effective intervention strategy or therapeutics are urgently needed for this deadly disease.

Tumor microenvironment has been known to play an essential role in regulating tumor growth and progression. The mode and status of the interaction between cancer cells and their surrounding constituents called stroma, including various types of cells (e.g. fibroblasts, vascular endothelial cells and immune cells) and extracellular molecules, is crucial in affecting tumor progression and metastasis [3, 4]. The interplay between cancer and stroma regulates the distribution pattern of immune cells in tumor microenvironment, which in turn control the tumor progression including initial colonization, immune escape and metastasis [5, 6]. Tumor-associated macrophages (TAMs) including type 1 (M1) and type 2 (M2) macrophages have anti- or pro-tumor functions, respectively, and play a fundamental role in innate and adaptive immune responses relevant to cancer cell growth [7]. TAMs with the M2 phenotype are associated with a poor prognosis in cancer patients, as compared to TAMs with the M1 phenotype [8, 9]. Moreover, macrophages with the M2 phenotype can degrade extracellular matrix (ECM), such as fibronectin and collagen I, thereby enhancing tumoral angiogenesis via release and mobilization of angiogenic factors [10–12]. Cancer-associated fibroblasts (CAFs), the other abundant tumor stromal cells, are the major source of ECM degradation proteases and contribute to epithelial ovarian carcinoma metastasis through promoting tumor cell invasion [13]. Further, normal peritoneal fibroblasts that are located in the tumor microenvironment can be activated to express CAF markers that enhance ovarian cancer cell and endothelial cells growth [14]. However, little is known how extracellular molecules secreted from cancer cells regulate stroma status to facilitate ovarian cancer progression. Targeting M2 macrophages is a plausible anti-cancer strategy. Likewise, targeting CAFs could be another therapeutic strategy against ovarian cancer and needs further study.

Periostin (POSTN), a secreted matricellular protein, involves in many fundamental biological events such as cell proliferation, tumor angiogenesis and metastasis [15, 16]. Upregulation of POSTN associates with increasing cell migration, chemoresistance and poor prognosis in various human cancers including ovarian cancer [17–24]. In an orthotopic mouse model of ovarian cancer where cancer cells are engineered to express POSTN resulted in potent tumor angiogenesis and metastasis [16]. Furthermore, POSTN also promotes tumor progression and metastatsis in other cancers such as head and neck squamous cell carcinoma, melanoma, colorectal carcinoma, lung cancer and breast cancer [25–29]. Targeting POSTN using its binding DNA aptamer or neutralizing antibody significantly inhibits breast and ovarian cancer cell metastasis [26, 28, 29]. A previous report showed that glioma stem cell-secreted POSTN can recruit M2-type TAM and the density of TAM correlates with POSTN expression level in glioblastoma multiformes (GBMs) [30]. They also found that either blocking POSTN by shRNA or integrin αvβ_3_ by inhibitory RGD peptide reduces TAM recruitment [30]. In ovarian cancer, high level of POSTN in ovarian cancer ascites fluids correlates with CD163 + TAMs infiltration and poor relapse-free survival in patients [31]. Co-culture of A2780 ovarian cancer cells with THP-1-derived macrophages increases the expression of POSTN and its secretion from ovarian cancer cells, which could also be induced by TGF-β secreted from macrophages [31]. It was shown that CAF-derived POSTN enhanced cancer stemness by activating protein tyrosine kinase 7-Wnt/β-catenin signaling in HNSCC [32]. Although the role of POSTN in regulating TAMs or CAFs has been discussed, the interplay among ovarian cancer, TAMs and CAFs in ovarian cancer progression, as well as the signaling pathway(s) that cancer-cell-derived POSTN regulates in ovarian cancer is still unknown.

Using isogenic pairs of low and highly invasive human ovarian cancer lines, we identified POSTN to be involved in regulating ovarian cancer cell growth and metastasis. Our data indicated that POSTN acted via integrin-dependent NF-κB and TGF-β2 signaling to induce production of cytokines/chemokines from cancer cells to promote mobilization and differentiation of M2 macrophages and activate CAFs in tumor microenvironment, resulting in enhanced growth and metastasis. We also found that elevated POSTN expression correlated with advanced stages of ovarian cancer and patient survival. Therefore, POSTN could be a useful prognosis marker and therapeutic target for epithelial ovarian cancer.

## Methods

### Cell culture and conditioned medium preparation

Human epithelial ovarian carcinoma cell lines A2780 and A1847 were gifts from Dr. Stuart Aaronson (Mount Sinai School of Medicine, NY, USA). The TOV-112D, SKOV3, IOSE and ES-2 cell lines were purchased from ATCC (Manassas, VA, USA). The SKOV-I6 and A2780-I4 cell lines were selected from the parental cell lines SKOV3 and A2780 by 6 or 4 times of in vitro invasion assays, respectively [33]. The OVS1 cell line was established from a serous type of human ovarian tumor obtained from Taipei Veterans General Hospital (TVGH), Taiwan [33–35]. IOSE cells were cultured in medium 199:MCDB 105 (1:1) medium contains 10% fetal bovine serum (FBS) (Sigma-Aldrich, St Louis, MO, USA). Cells except IOSE, were grown in Dulbecco's Modified Eagle’s Medium (DMEM), supplemented with 10% FBS and penicillin/streptomycin (Invitrogen, Waltham, MA, USA). Human monocytic leukaemia THP-1 cells were grown in RPMI 1640 medium, supplemented with 10% FBS and penicillin/streptomycin (Invitrogen). Normal human adipose derived stromal cells (hADSC) were a gift from Dr. Chang Cheng-Chi, Graduate Institute of Oral Biology, National Taiwan University, Taipei, Taiwan. hADSCs were grown in DMEM/F12 (1:1) medium (Invitrogen) and supplemented with 10% FBS and 1 ng/mL basic fibroblast growth factor (R&D Systems, Minneapolis, MN, USA). All the cells were maintained in the exponential growth phase at 37° C in 5% CO_2_. For collection of conditioned medium, 1 × 10^6^ cells were seeded in a 10-cm culture dish with DMEM and 10% FBS overnight. Next day, the culture medium was substituted with RPMI 1640 medium containing 0.5% FBS for an additional 24 h. The conditioned medium was collected and filtered through a low protein binding 0.45-μm polyvinylidene fluoride (PVDF) filter (Millipore, Burlington, MA, USA) and stored in a – 80 °C freezer before use. Reagents used in this study are listed in Additional file 1: Table S1.

### Exon array analysis

Briefly, total RNA of cell lines were extracted, and analyzed by Affymetrix Human Exon 1.0 ST Array (Affymetrix, Santa Clara, CA, USA). The raw data was normalized and analyzed by GeneSpring software (Agilent Technologies, Santa Clara, CA, USA), followed by Gene Ontology annotation analysis, and classified into GO molecular function domains. Genes that expression changes greater than 1.5 fold were selected for heatmap analysis. The heatmap was plotted in R with pheatmap package (Lucent Technologies, Murray Hill, NJ, USA). Color scale bar indicates log2 fold change after column normalization.

### Public domain data analysis

The sources of gene expression profiling and clinical pathological characteristics of Tothil cohort (GSE9891) were downloaded from Oncomine (www.oncomine.org). The median of *POSTN* expression levels were used as cut off points for the overall survival analysis.

### Gene expression manipulation

For overexpression experiments, *POSTN* cDNA was subcloned into the pcDNA4 mammalian expression vector (Invitrogen) and was transfected into low POSTN expressing cells. POSTN stably expressing cells were maintained in medium containing zeocin. For knockdown experiments, the high POSTN expressing cells were infected with pLKO.1-*POSTN* or control pLKO.1-*Lu*c lentiviral vector (National RNAi core facility, Academia Sinica, Taipei, Taiwan) and were maintained in the complete medium containing puromycin. siRNA and shRNA clones used in this study are listed in Additional file 1: Table S2.

### Western blot analysis

Total cell lysates were prepared in RIPA lysis buffer supplemented with protease inhibitor and phosphatase inhibitor cocktails. Proteins were separated by SDS-PAGE, transferred onto the PVDF membrane and non-specific binding was blocked 1 h in 5% skim milk. Proteins were detected by applying respective specific primary antibody for 1 h, followed by incubation with horseradish peroxidase-conjugated secondary antibody for 1 h at room temperature. The chemiluminescence system was used to visualize the signals. Antibodies against specific proteins in this study are listed in Additional file 1: Table S3.

### Migration and invasion assays

The in vitro migration and invasion assays were performed using Transwells (Corning, Corning, NY, USA). For the cancer cell migration assay, 2.5 × 10^4^ cells were seeded in uncoated Transwells, with 8.0 μm pores (Corning). For the invasion assay, 1 × 10^5^ cells were seeded in a Matrigel-coated chamber with 8.0 μm pores (BD Bioscience, Franklin Lakes, NJ, USA). The migrated or invaded cells in polycarbonate membranes were stained and counted using previously described methods [36, 37]. For THP-1 monocytic cell migration assay, 2.5 × 10^5^ cells were seeded onto the inserts with a porous PET (Corning) membrane (pore size, 8.0 μm) in 350 μL of RPMI 1640 supplemented with 0.5% FBS. The inserts were placed in the wells, which contained cancer cell-derived conditioned medium, and incubated for 6 h at 37 °C. The migration of the THP-1 cells was visualized under a microscope and quantified by counting three randomized 100 × fields of the migrated cells on the underside of insert.

### Quantitative RT-PCR analysis

The qRT-PCR was performed as described previously [36]. Total RNA from the cultured cells was extracted using TRIzol reagent (Invitrogen) following the protocols recommended by the manufacturer. First-strand cDNA was generated by the SuperScript^®^ III First-Strand Synthesis System (Invitrogen) using oligo-dT primer. The KAPA SYBR FAST Universal qPCR Kit (KAPA Biosystems, Wilmington, MA, USA) was used for gene detection in a CFX96 real-time PCR detection system (Bio-Rad, Hercules, CA, USA). The 2^−∆CT^ or 2^−∆∆CT^ method was used to calculate the relative expression of specific genes. The expression level of the β-actin (ACTB) housekeeping gene was used as an internal control. The primer sequences are listed in Additional file 1: Table S4. The annealing temperature for all the primer pairs was 60 °C.

### ELISA assay

The expression level of secreted TGF-β1 or TGF-β2 was detected by using human TGF-beta1 ELISA kit (#ELH-TGFb1) or human TGF-beta2 ELISA kit (#ELH-TGFb2-1) (RayBiotech, Peachtree Corners, GA, USA) according to user manual. Briefly, standard or sample were added to each pre-coated well and incubate 2.5 h at room temperature with gentle shaking. Add biotinylated antibody, streptavidin solution and finally add TMB One-Step substrate reagent for color visualization. The protein levels of TGF-β1 or TGF-β2 were detected under 450 nm by ELISA reader.

### Clinical samples

Ovarian tumor RNA samples were obtained from the Department of Obstetrics and Gynecology, China Medical University Hospital, Taichung, Taiwan, with the approval of the institutional review board (IRB#CMUH107-REC1-095). Informed written consent was obtained from all the patients who participated in the study. Tumor samples were collected during debulking surgery. The identities of the patients from whom the pathological specimens were obtained remained anonymous. In addition, a commercial ovarian cancer cDNA was purchased from OriGene (Rockville, MD, USA) and used in this study. Detail information was summarized in Additional file 1: Tables S5 and S6.

### In vivo xenograft studies

Animal experiments were conducted as previously described [33]. Three xenograft models were used in this study: subcutaneous (s.c.), intraperitoneal (i.p.) and orthotopic metastasis models. In the subcutaneous and intraperitoneal models, SKOV3-derived cells (1 × 10^6^) were harvested and resuspended in 100 μL of PBS. Tumor xenografts were established by injecting SKOV3-derived cells into the dorsal flank (s.c.) or abdominal cavity at right lower quadrant (i.p.) of mice. In the orthotopic metastasis model, SKOV3 or its derived highly invasive subline SKOV-I6 cells (1 × 10^6^) were resuspended in 20 μL of PBS containing 50% Matrigel (BD Biosciences) and intra-bursally injected into ovary capsules of the mice. Female NOD/SCID mice (aged 6–8 weeks, National Laboratory Animal Center, Taipei, Taiwan) were randomly assigned to each experimental group. For the s.c. tumor growth model, the dimensions of the xenografts were measured by callipers every week and tumor volume was calculated using the formula V = (π × length × width^2^)/6. For the i.p. model, tumor growth and abdominal metastases were monitored by in vivo imaging system (PerkinElmer, Waltham, MA, USA). The mice were sacrificed 4 weeks (s.c. and i.p. models) or three weeks (orthotopic metastasis model) after inoculation of the tumor cells. All the xenografts were fixed in 10% neutral buffered formalin, embedded in paraffin and cut consecutively into 4-µm sections for further pathological examination after hematoxylin and eosin staining and for subsequent immunohistochemistry analysis.

### FACS analysis

For the FACS analysis, the differentiated M1 and M2 macrophages were collected and stained with anti-CD68 (eBioscience, Waltham, MA, USA), anti-CD206 (eBioscience) or anti-CD80 (eBioscience) antibody following the protocols recommended by the manufacturer. Antibodies against specific proteins in this study are listed in Additional file 1: Table S4.

### Immunohistochemistry and immunofluorescence

Briefly, paraffin-embedded ovarian cancer tissue sections (4 μm) on poly-l-lysine-coated slides were deparaffinized and rinsed with 10 mM Tris–HCl (pH 7.4) and 150 mM sodium chloride. Peroxidase was quenched with methanol and 3% hydrogen peroxide. The slides were then placed in 10 mM citrate buffer (pH 6.0) at 100 °C for 20 min in a pressurized heating chamber. After incubation with POSTN (1:100), F4/80 (1:100), CD206 (1:500) or α-SMA (1:500) primary antibodies individually for 1 h at room temperature, the slides were thoroughly washed three times with PBS. Bound antibodies were detected using the EnVision Detection Systems Peroxidase/DAB, Rabbit/Mouse kit (Dako, Glostrup, Denmark). The slides were then counterstained with haematoxylin. Finally, the slides were photographed under a microscope (BX50, OLYMPUS, Tokyo, Japan). Negative controls were obtained by performing all the steps but omitting the primary antibodies. For immunofluorescence staining, cells were fixed with 4% paraformaldehyde/PBS for 30 min followed by permeabilizing cells with 0.5% Triton X-100/PBS for 15 min at room temperature. Nonspecific binding sites were blocked using 1% BSA/PBS for 1 h. Primary specific antibody was applied for 1 h, followed by fluorophore-conjugated secondary antibody incubation for additional 1 h. Cells were washed with 0.1% Tween 20/PBS (PBST) and were mounted with anti-fading agent. Images were captured under Leica TCS SP5 (Leica Microsystems, Wetzlar, Germany) and were processed using LASX software (Leica Microsystems). Antibodies against specific proteins in this study are listed in Additional file 1: Table S3.

### Proximity ligation assay (PLA)

The PLA was applied for in situ detection of endogenous protein–protein interaction between POSTN and integrin β3 or POSTN and integrin β5. This assay was carried out as described in user manual (Duolink^®^ PLA DUO92008, Sigma-Aldrich). In brief, cells were seeded on glass coverslips overnight then fixed by ice-cold 100% methanol for 15 min at − 20 °C. Aspirated fixative, rinsed three times in PBS then added blocking solution to samples for 30 min at 37 °C. Primary antibodies anti-POSTN antibody (SC-46655, Santa Cruz Biotechnology, Dallas, TX, USA), anti-integrin β3 antibody (#13166, Cell Signaling Technology, Danvers, MA, USA) and anti-Integrin β5 antibody (#3629; Cell Signaling Technology) were diluted 1:100 in buffer and added to samples for overnight at 4 °C. Washed in PBST then followed by applying PLA plus and minus probes to samples for 1 h at 37 °C. Washed in PBST twice. Ligation was performed by ligase application for 30 min at 37 °C, followed by amplification with polymerase for 2 h at 37 °C. Washed samples with 2 × SSC twice then 0.2 × SSC once. Mounted samples with mounting media with DAPI. Signal was detected by confocal microscopy.

### In vitro co-culture model

Co-culture of THP-1 with ovarian cancer cells was done in RPMI 1640 supplemented with 10% FBS in 6-well cell culture inserts with a permeable PET membrane (pore size, 0.4 μm). The tumor cells were seeded at a density of 2 × 10^5^ cells per insert in 1.5 mL of medium. The THP-1 cells were seeded at a density of 2 × 10^5^ cells in the lower compartment in 3 mL of medium. After co-culturing for 5 days, the THP-1 cells were harvested for FACS analysis, RNA extraction and qRT-PCR analysis.

### Statistical analysis

Kaplan–Meier analysis using the *p* value of the log-rank test was applied to determine the power of POSTN as a marker for overall patient survival. All data are the mean ± SD, unless otherwise specified. The means ± SD represent data from three independent experiments. One-way ANOVA was used to compare the means among three or more independent groups. Student’s *t*-test was used to compare the means between two groups to be compared. A *p* value of less than 0.05 was considered statistically significant.

## Results

### Identification of POSTN to be potentially involved in ovarian cancer cell growth and invasion

**Fig. 1:**
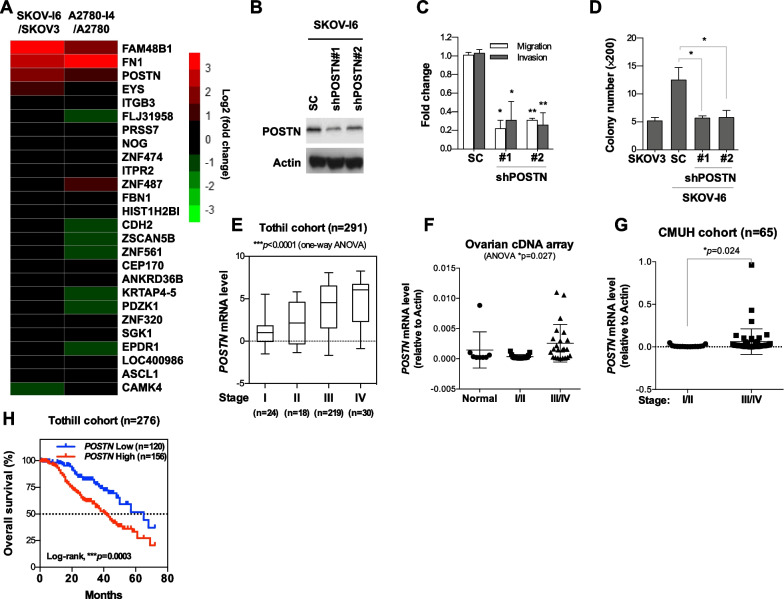
POSTN enhances migratory, invasive and colony forming abilities of ovarian cancer cells in vitro. **A** Heatmap of gene transcripts expression changes that are greater than 1.5 fold in both A2780 and SKOV3 isogenic high/low invasive cell line pairs by microarray. **B** Western blot analysis for detecting the POSTN level in SKOV-I6 cells infected with lentiviral vectors encoding shPOSTN or a scrambled control. **C** Cell migration and invasion was performed using 8.0-μm pore chamber for 15 h. A non-specific shRNA was used as the control. *p < 0.05, **p < 0.01. **D** Anchorage independent growth in soft agar of SKOV3 and SKOV-I6 cells with or without POSTN knockdown was evaluated by seeding 1 × 10^4^ cells in 6-well plates containing 0.4% low melting agar and culturing for 7 days. **E**–**G** POSTN was correlated with advanced-stages of ovarian cancer in Tothill cohort (GSE9891) and commercial ovarian cancer cDNA array and CMUH datasets. **H** Kaplan–Meier curves were generated to assess correlations between POSTN expression and 5-year survival rates in ovarian cancer patients from the Tothill (GSE9891) (n = 276) datasets. Median value was used as the cut off for grouping low and high POSTN expression patients

We have previously established several isogeneic pairs of low and highly invasive ovarian cancer cell lines [33, 34]. To search for putative genes that may play important roles in ovarian cancer progression and metastasis, we compared the gene expression profiles of highly invasive ovarian cancer lines A2780-I4 and SKOV-I6 with their parental low invasive lines A2780 and SKOV3, respectively, by exon microarray analysis. From the differentially upregulated genes of greater than 1.5 fold, we identified those that were overlapping in the two isogenic pairs, and thus were more likely to be involved in cancer invasion and metastasis (Fig. 1A; Additional file 1: Fig. S1A). The first criterion for being a candidate gene to be pursued is its elevated expression in both SKOV-I6 and A2780-I4 cells compared with their parental cells. FAM48B1, FN1 and POSTN were identified by this criterion. However, the biological function of FAM48B1 is rather unclear, and FN1 has been quite well characterized, so we decided to focus on evaluating the role of POSTN in ovarian cancer progression and metastasis.

### POSTN promotes growth, migration and invasion of ovarian cancer cells in vitro

We have evaluated the endogenous POSTN expression level in various ovarian cells (Additional file 1: Fig. S1A). To investigate whether POSTN modulated ovarian cancer cell migration and invasion, the SKOV-I6 cells were transduced with lentiviral vectors encoding shRNA against POSTN (shPOSTN) or scrambled control (SC) and followed by in vitro invasion/migration assays. The results showed that the POSTN-silenced SKOV-I6 cells had significantly decreased invasion and migration abilities than the controls cells (Fig. 1B, C). We also ectopically expressed POSTN in IOSE, TOV-112D and SKOV3 cells followed by migration and invasion assay of the cells. The results showed that ectopic expression of POSTN in those cells significantly increased their migration and invasion abilities compared to those of the controls (Additional file 1: Fig. S1B). Knockdown of POSTN in SKOV-I6 and OVS1 cells resulted in reduced growth in soft agar, as compared to the shRNA control transfected cells (Fig. 1D; Additional file 1: Fig. S1C). POSTN overexpression in TOV-112D cells resulted in increased colony formation in soft agar (Additional file 1: Fig. S1D). In addition to affecting migration and invasion ability, we observed knockdown of *POSTN* led to decreased adhesion ability of cancer cells, an important property for peritoneal metastasis of ovarian cancer cells (Additional file 1: Fig. S1E). These data indicate that POSTN regulates ovarian cancer cell growth, migration, invasion and adhesion ability.

### *POSTN* expression levels positively correlate with the cancer stages and are associated with poor survival of ovarian cancer patients

To explore the clinical relevance of POSTN, we analysed its expression from the public domain datasets, and clinical samples from commercial company or Department of Obstetrics and Gynecology, China Medical University Hospital, Taiwan. We found that the expression of *POSTN* was higher in the advanced stage ovarian tumors (Fig. 1E–G; Additional file 1: Fig. S1F–H). Kaplan–Meier survival analysis showed that patients with higher *POSTN* expression had a poorer overall survival than those with low *POSTN* expression (Fig. 1H). These data indicate that *POSTN* may serve as a prognostic biomarker and therapeutic target for ovarian cancer.

### POSTN regulates integrin-dependent ERK and NF-κB signaling pathways in ovarian cancer cells

**Fig. 2:**
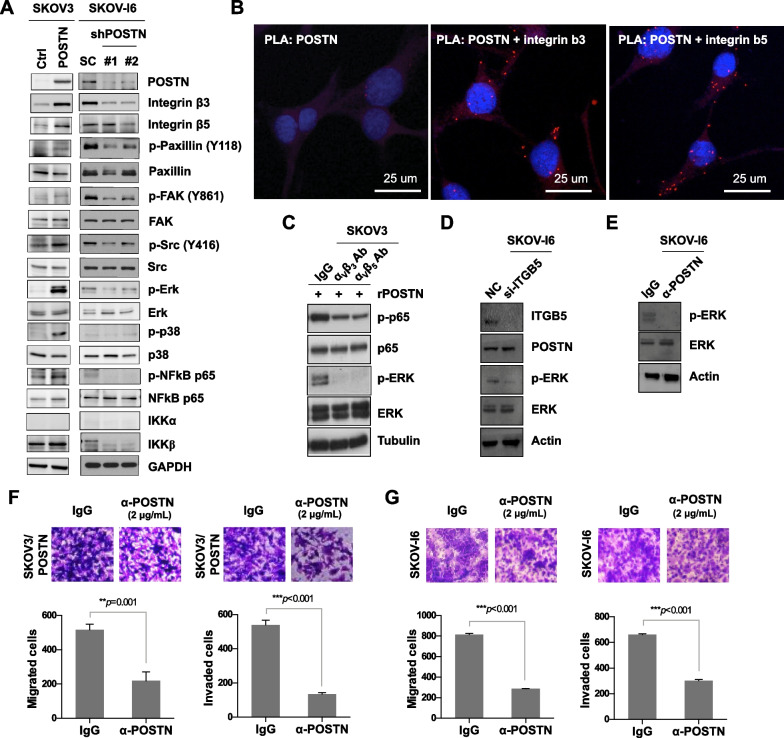
POSTN regulates the ERK/NF-κB axis to modulate ovarian cancer cell migration and invasion. **A** Western blot analysis for detecting phosphoproteins and proteins as indicated in SKOV3 cells transfected with POSTN or in SKOV-I6 cells infected with lentiviral vectors encoding shPOSTN or a scrambled control. **B** PLA images show protein–protein interactions between POSTN and integrin β3 or integrin β5 in SKOV-I6 cells. The scale bar represents 25 μm. **C** Western blot analysis for detecting phosphoproteins and proteins as indicated in SKOV3 cells treated with recombinant POSTN protein (100 ng/mL), in combination with anti-α_v_β_3_ (10 μg/mL) or anti-α_v_β_5_ (10 μg/mL) neutralizing antibody as indicated. **D** Western blot analysis for measuring proteins and phosphoprotein as indicated in SKOV-I6 cells transfected with siITGB5 or control oligonucleotides. **E** Western blot analysis for detecting p-ERK and ERK in SKOV-I6 cells treated with antibody against POSTN or IgG. **F**, **G** Migration and invasion assays of SKOV3/POSTN or SKOV-I6 cells treated with a specific POSTN monoclonal antibody (2 μg/mL) or IgG. **p < 0.01; *** *p* < 0.001

Previous studies suggested that various factors, such as ovulation, endometriosis and pelvic inflammatory diseases were associated with inflammation of the OSE, and posted an increased risk of EOC. Research also demonstrated that NF-κB activation was frequently associated with an inflammatory microenvironment during malignant progression [38, 39]. Among the main transcription factors involved in inflammation, NF-κB is the most important one in regulating chronic inflammatory diseases [40]. Further, the expression of POSTN was positively correlated with that of NF-κB in ovarian tumors from patients (Additional file 1: Fig. S2A). To determine whether POSTN could mediate NF-κB activation in ovarian cancer cells, we examined the expression and phosphorylation status of proteins associated with the PI3K and NF-κB signaling pathways. The levels of p-ERK, p-p38, IKKβ and p-p65, but not that of IKKα, were increased in the SKOV3, IOSE or ES-2 cells transfected with the pcDNA4/POSTN expression plasmid when compared with the cells transfected with the pcDNA4 control plasmid (Fig. 2A; Additional file 1: Fig. S2B). Furthermore, POSTN knockdown significantly reduced the levels of p-ERK, p-p38, IKKβ and p-p65 in the SKOV-I6 cells (Fig. 2A). The results of PLA experiment showed the protein–protein interactions between POSTN and integrin β3 or integrin β5 in the SKOV-I6 cells (Fig. 2B). Also, POSTN colocalized with integrin β3 or integrin β5 in SKOV-I6 cells (Additional file 1: Fig. S2C, D). As reported previously, POSTN is a secreted protein, which can bind to α_v_β_3_ and α_v_β_5_ integrins on ovarian cancer cells [17]. To further validate the binding specificity of POSTN and integrins in the SKOV3 cells, the cells were pre-treated with α_v_β_3_ or α_v_β_5_ integrin neutralizing antibodies followed by addition of recombinant POSTN. The results showed that blocking integrins with their neutralizing antibodies decreased POSTN-induced p65 and ERK phosphorylation in SKOV3 cells (Fig. 2C). In addition, p-ERK activation was reduced upon treatment of SKOV-I6 cells with integrin β5 (ITGB5) siRNA or POSTN antibody, respectively. (Fig. 2D, E). Treatment with a POSTN monoclonal antibody attenuated migratory and invasive abilities of SKOV3/POSTN, OVS1 and SKOV-I6 cells (Fig. 2F, G; Additional file 1: Fig. S2E). These results suggest that activation of ERK/NF-κB signaling pathway correlates with POSTN promoted migration and invasion.

### POSTN promotes ovarian cancer growth and metastasis in vivo

**Fig. 3:**
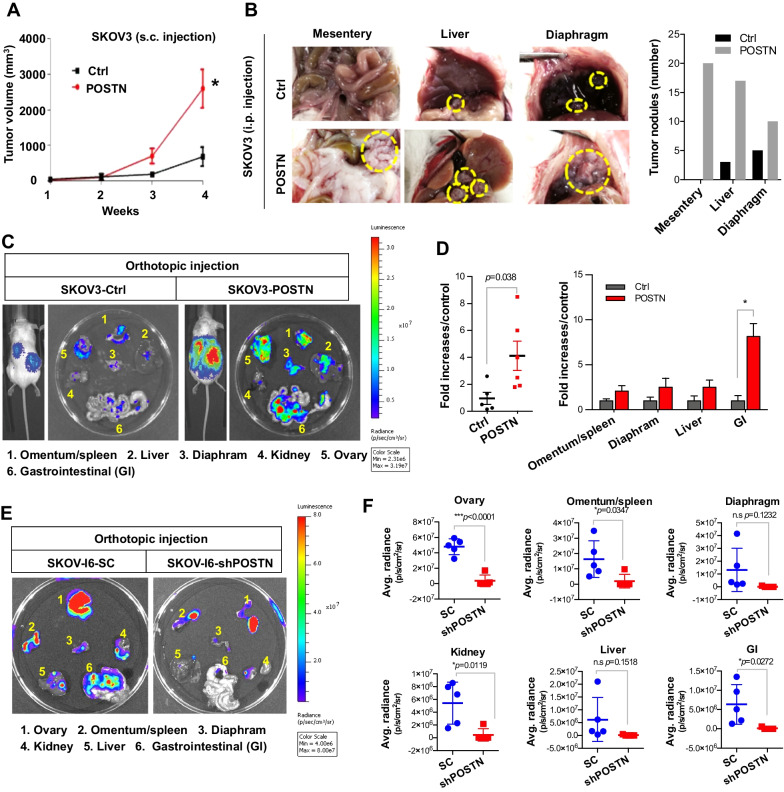
POSTN expression promotes ovarian tumor growth and metastasis in vivo. **A** SKOV3 cells were stably transfected with a control or a POSTN-expressing plasmid and inoculated into SCID mice s.c. for 4 weeks (*n* = 5 mice in each group). The volume of the s.c. xenograft tumors was measured each week. *p < 0.05. **B** POSTN overexpression enhanced the growth of SKOV3 cells in the i.p. mouse model (*n* = 6 mice in each group). Representative images of xenograft nodules are indicated by yellow circles (left). Quantification results of nodules are also indicated (right). **C** Control or POSTN-expressing vector was transfected into SKOV3 cells containing stable luciferase expression plasmid and orthotopically injected into ovary capsules of the mice. The kinetics of abdominal metastases was monitored by BLI. **D** Histogram of quantitative BLI signals showed the abdominal or specific organ metastasis of SKOV3/POSTN-bearing mice comparing with control mice. **E** Representative BLI images showed the metastasis potential of SKOV-I6/SC or SKOV-I6/shPOSTN by BLI. **F** The quantitative data from **E**. *p < 0.05; ***p < 0.001

Based on the results in Fig. 1, POSTN was able to promote ovarian cancer cell growth and invasion in vitro*.* We next investigated the effect of POSTN on ovarian tumor growth and metastasis. The tumor mass was significantly bigger with injection of the SKOV3/POSTN as compared to the SKOV3/pcDNA4 control cells (1 × 10^6^ cells per mouse) in a s.c. xenograft model (Fig. 3A). In the i.p. injection model, more tumors were observed in the mesentery, liver and diaphragm four weeks after the injection of SKOV3/POSTN cells at 1 × 10^6^ cells per mouse compared to the SKOV3/ pcDNA4 control (Fig. 3B). In the orthotopic implantation and metastasis model, the bioluminescent imaging (BLI) data indicated that the mice implanted with the SKOV3/POSTN cells (also at 1 × 10^6^ cells per mouse) had a significantly higher number of abdominal metastases at various sites than the mice implanted with the control SKOV3/pcDNA4 cells (Fig. 3C, D). We also orthotopically injected *POSTN-*silenced SKOV-I6 or scrambled control SKOV-I6 cells followed by monitoring the metastasis potential via BLI. The ex vivo data showed *POSTN* knockdown reduced the metastatic ability to the peritoneal sites such as omentum/spleen, kidney and gastrointestinal regions (Fig. 3E, F; Additional file 1: Fig. S3). These results indicate that POSTN promotes ovarian cancer cell growth and metastasis in vivo.

### POSTN attracts monocytes infiltration and promotes M2 macrophages polarization

**Fig. 4:**
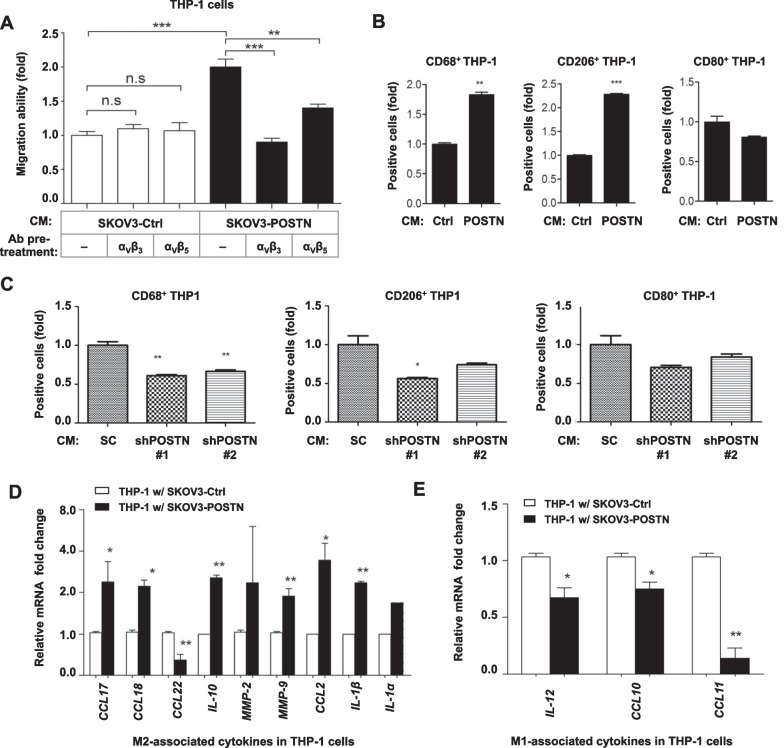
POSTN increases monocytes migration and promotes M2 macrophages polarization. **A** THP-1 cell migration ability was determined by in vitro migration assay upon addition of the control SKOV3/pcDNA4 or SKOV3/POSTN conditioned medium with or without specific neutralizing antibody pre-treatment in the bottom well. The α_v_β_3_ or α_v_β_5_ integrin neutralizing antibody, as indicated, was added before collecting the conditioned medium. *p < 0.01, ***p < 0.001. **B** Flow cytometry analysis of THP-1 cells treated with the vector control or POSTN-overexpressing conditioned medium to measure CD68-positive, CD206-positive or CD80-positive populations (left, middle and right, respectively). **p < 0.01, ***p < 0.001. **C** Flow cytometry analysis of THP-1 cells treated with conditioned medium from the non-specific (N/S) shRNA control or shPOSTN SKOV-I6 cells to measure CD68-positive, CD206-positive or CD80-positive populations (left, middle and right, respectively). *p < 0.05, **p < 0.01. **D**, **E** qRT-PCR analysis of THP-1 cells co-cultured with SKOV3 cells transfected with a control or POSTN-overexpressing vector for 5 days to measure M2- or M1-associated cytokines expression change (**D**, **E**, respectively). The mRNA expression levels were compared based on the relative fold-change of each gene and the associated *p* values were calculated using a paired *t* test. *p < 0.05, **p < 0.01

We further analysed whether conditioned medium from ovarian cancer cells had a chemotactic effect on THP-1. Conditioned medium from the POSTN-overexpressing SKOV3 cells had a significantly higher chemotactic effect on THP-1 cells compared with that from control cells in a Boyden chamber assay (Fig. 4A). The direct effect of POSTN on monocyte migration and polarization was also evaluated by adding THP-1 cells with recombinant POSTN (rPOSTN). The results showed rPOSTN directly promotes migration and M2 macrophage polarization of THP-1 cells (Additional file 1: Fig. S4A). The addition of α_v_β_3_ or α_v_β_5_ integrin neutralization antibody to the conditioned medium significantly reduced its chemotactic effect (Fig. 4A). Our result suggested that enhanced chemotactic effect on THP-1 cells was dependent on direct effect of POSTN or certain soluble factors induced by POSTN. Such function was α_v_β_3_ and α_v_β_5_ dependent. The results of flow cytometry analysis indicated that the conditioned medium from SKOV3/POSTN cells induced monocytic cell differentiation into M2 macrophages (CD206^+^) but not M1 macrophages (CD80^+^) (Fig. 4B). Further, expression of CD206 in THP-1 cells was also induced after rPOSTN treatment (Additional file 1: Fig. S4B). The conditioned medium from SKOV-I6/shPOSTN cells had significantly reduced activity to induce the expression of CD68 and CD206 in THP-1 cells (Fig. 4C). Consistently, the mRNA levels of M2 macrophage-associated cytokines including *CCL17, CCL18, IL-10, MMP-9, CCL2* and *IL-1β* were significantly elevated when THP-1 cells were co-cultured with SKOV3/POSTN cells, in comparison with co-culturing with the control cells (Fig. 4D). In contrast, M1 macrophage-associated cytokines, including *IL-12*, *CCL10* and *CCL11*, were repressed in THP-1 cells co-cultured with the SKOV3/POSTN cells (Fig. 4E). These results suggest that POSTN released by ovarian cancer cells promotes integrin-dependent monocytes chemotaxis and M2 macrophage polarization in direct or indirect manner, and thus facilitating the evasion of immunosurveillance and promotion of tumor progression.

### POSTN expression enriches TAMs infiltration and promotes M2 macrophage -associated chemokines expression in vivo

**Fig. 5:**
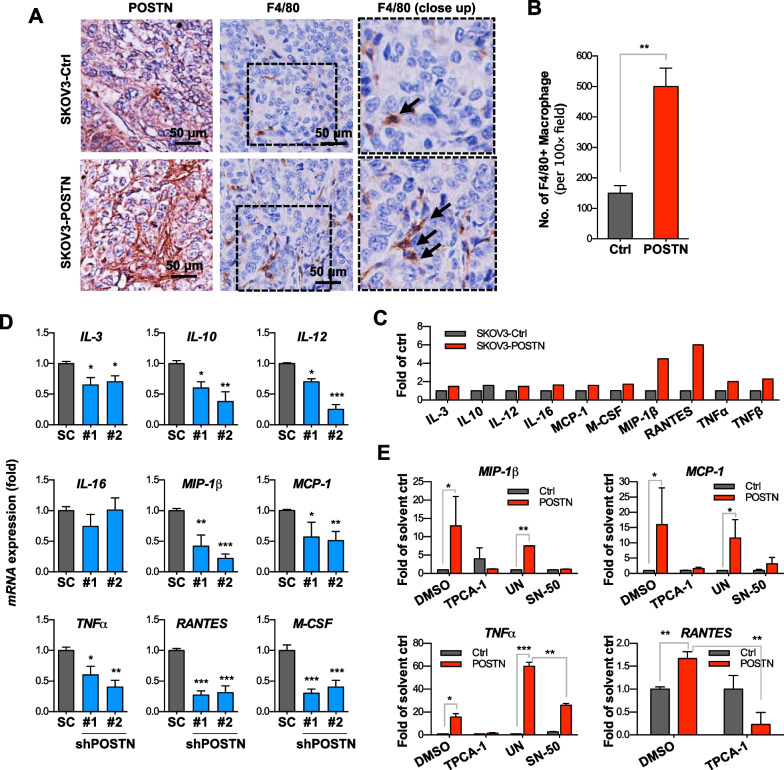
POSTN-overexpressing SKOV3 cells induces chemotactic cytokines expression in vitro and increases TAM in xenograft tumors. **A** Immunohistochemistry staining of POSTN and murine F4/80 (a marker of macrophages) molecules with respective antibodies using orthotopic xenografts tumor sections derived from SKOV3 cells transfected with control or POSTN-expressing vector. Scale bars = 100 μm. **B** Quantification of F4/80 staining intensity by ImageQuant under microscopy. **p < 0.01. **C** Conditioned media were collected from SKOV3 cells stably transfected with pcDNA4 (Ctrl) or pcDNA4/POSTN (POSTN) for cytokine array analysis. The cytokine expression was quantified by Image J software. **D** Cytokine mRNA expression was measured by qRT-PCR in SKOV-I6 cells transfected with either of the lentivirus-delivered shRNAs (shRNA#1 and shRNA#2) plasmid. Non-specific shRNA was used as the control (SC). **E** The effect on cytokine mRNAs production was detected by qRT-PCR after treatment with TPCA-1, an inhibitor of the NF-κB pathway (1 μM) or SN-50 (18 μM), an inhibitor against NF-κB nuclear translocation, for 48 h in control (white bars) or POSTN-overexpressing SKOV3 cells (black bars). DMSO (0.1 μL/mL) or water (solvent for SN50, labelled as UN) was used as the control. The bar graph represents the mean fold change ± SD from three independent experiments

Based on the above in vitro observations, we then examined whether POSTN played a role in modulating tumor microenvironmental compartments, especially TAMs. Using an orthotopic xenograft model, we observed that SKOV3/POSTN cells-derived tumors harboured greatly enriched TAMs in the stroma as determined by immunostaining with the antibodies recognizing murine macrophage markers F4/80 (Fig. 5A, B). This is consistent with the findings of a previous study in GBM [30]. We then explored the underlying mechanism by checking if POSTN could affect the expression of cytokines/chemokines involved in macrophages mobilization and maturation. Cytokine array assay results showed that POSTN overexpression in SKOV-I6 cells induced protein expression of several cytokines and chemokines, including IL-3, IL-10, IL-12, IL-16, monocyte chemoattractant protein-1 (MCP-1), MCSF, MIP1-β, RANTES, TNF-α and TNF-β (Fig. 5C). Conversely, *POSTN* knockdown reduced the expression of those cytokine mRNAs (Fig. 5D). SN50, an inhibitor against NF-κB nuclear translocation, was able to inhibit POSTN-induced cytokine production in the ovarian cancer cells (Fig. 5E). Similarly, an IKKβ inhibitor, TCPA-1, reduced cytokine expression in the POSTN-overexpressing cancer cells (Fig. 5E). Together, these results suggest that POSTN facilitates attraction of monocytes and differentiation into TAMs by directly or inducing the expression of NF-κB-dependent cytokines and chemokines from cancer cells.

### POSTN modulates CAF activation through TGF-β2

**Fig. 6:**
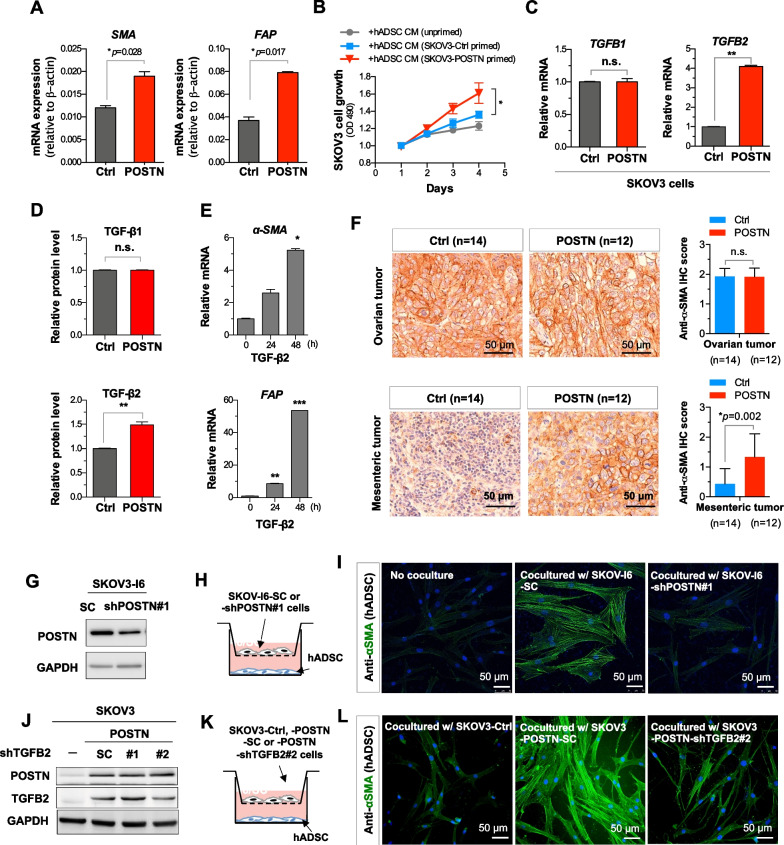
POSTN increases expression of TGF-β2 capable of inducing CAF markers and is correlated with abundant CAFs in tumor microenvironment. **A** The qRT-PCR analysis of α-SMA and FAP mRNAs from conditioned medium treated hADSCs. The conditioned media were collected from SKOV3/ctrl or SKOV3/POSTN cells cultured for 2 days. **B** MTT proliferation assay of SKOV3 cells incubated with conditioned medium from unprimed, SKOV3/ctrl cells or SKOV3/POSTN cells primed hADSC cells. **C**, **D** RNA and protein levels of TGF-β1 and TGF-β2 in POSTN-overexpressing SKOV3 cells were determined qRT-PCR and ELISA, respectively. **E** Expression levels of α-SMA and FAP transcripts in TGF-β2 treated normal human adipose derived stromal cells (hADSCs) were determined by qRT-PCR. **F** Representative immunohistochemical analysis for the expression of α-SMA in POSTN-overexpressing versus control SKOV3-derived primary and metastatic tumors in the orthotopic metastasis mouse model (left panel). Quantification results of the immunohistochemical analysis of anti-α-SMA (right panel). Six female NOD/SCID mice were randomly assigned to each group and were followed up for 3 weeks in orthotopic metastasis mouse model. **G** Western blot analysis for measuring the POSTN level in SKOV-I6 cells infected with lentiviral plasmid encoding shPOSTN or a scrambled control. **H** The diagram illustrates the co-culture system used for cancer cell-induced CAF markers activation. **I** Representative immunofluorescence images showe the α-SMA levels of hADSC in non-co-cultured (left), co-cultured with SKOV-I6/SC (middle) or co-cultured with SKOV-I6/shPOSTN#1 cells (right). Scale bar = 50 μm. **J** Western blot analysis for detecting TGFB2 and POSTN in POSTN overexpressing SKOV3 cells infected with lentiviral vector shTGFB2 or a scrambled control. **K** The diagram illustrates the co-culture system used for cancer cell-induced CAF markers activation. **L** Representative immunofluorescence images showing the α-SMA levels of hADSC in co-cultured with SKOV3/ctrl (left), SKOV3/POSTN-scrambled control (middle) or SKOV3/POSTN-shTGFB2#2 cells (right). *p < 0.05; **p < 0.01; ***p < 0.001

In addition to TAMs, CAFs also play an important role in promoting tumor progression in tumor microenvironment. To evaluate the involvement of POSTN in modulating CAF activation, we surveyed the expression levels of CAF markers such as alpha smooth muscle actin (α-SMA) and fibroblast activation protein alpha (FAP) in the POSTN-high and POSTN-low ovarian cancer patients from the TCGA database. We observed both α-SMA (Additional file 1: Fig. S5A) and FAP (Additional file 1: Fig. S5B) were highly expressed in POSTN-high sub-populations, implying POSTN may be associated with increasing abundance of CAFs. To assess the involvement of POSTN in CAF activation in ovarian cancer, conditioned medium from POSTN-overexpressing or control SKOV3 cells were used for incubating normal hADSC for 5 days followed by determining the CAF marker genes expression. The results showed that conditioned medium from POSTN overexpressing SKOV3 cells induced higher *α-SMA* and *FAP* expression in hADSC cells (Fig. 6A; Additional file 1: Fig. S5C). Moreover, we compared the effect of conditioned medium from unprimed hADSC, SKOV3 primed hADSC and SKOV3/POSTN primed hADSC in promoting the growth of SKOV3 cells. The results showed that the SKOV3/POSTN primed hADSC conditioned medium had the highest activity (Fig. 6B; Additional file 1: Fig. S5C). Although several signaling pathways have been reported to be related to CAF activation such as TGF-β, PI3K/AKT/mTOR-activated protein kinase, Wnt and JAK [41, 42]. Among them, TGF-β is the well know growth factor for fibroblast activation. The epithelial cell-derived POSTN has been reported to induce TGF-β activation in human bronchial epithelial cell [43]. To explore the potential role of POSTN in CAF activation in ovarian cancer, we focused on examined the possible regulation of TGFβ, which is known to promote CAFs, by POSTN. We overexpressed POSTN in SKOV3 cells followed by qRT-PCR analysis for the expression of TGF-β2. The results showed that POSTN induced TGF-β2, but not TGF-β1, mRNA and protein expression in ovarian cancer cells (Fig. 6C, D). We further observed that TGF-β2 was able to induce the expression of CAF markers α-SMA and FAP in hADSCs (Fig. 6E). In the orthotopic model, immunohistochemical results showed SKOV3/POSTN cells-derived metastasized mesenteric tumors had significantly increased α-SMA expression compared to the control, however, no significant difference was observed for the primary tumors (Fig. 6F). We also found that knockdown of POSTN in SKOV-I6 ovarian cells, which had elevated POSTN expression, reduced α-SMA expression in hADSC in the co-culture system (Fig. 6G–I). To further assess the involvement of POSTN-*TGFB2* axis in regulating CAF activation, we overexpressed *POSTN* and simultaneously knocked down *TGFB2* in SKOV3 cells followed by co-culturing with hADSCs. Knockdown of *TGFB2* (the clone 2; siTGFB2#2) in POSTN overexpressing SKOV3 cells resulted in reduced α-SMA expression level in hADSC co-culture (Fig. 6J–L). The results suggest that autocrine effect of POSTN induces TGF-β2 expression from ovarian cancer cells to promote activation of normal stromal fibroblasts to become CAFs in metastatic tumors.

## Discussion

**Fig. 7:**
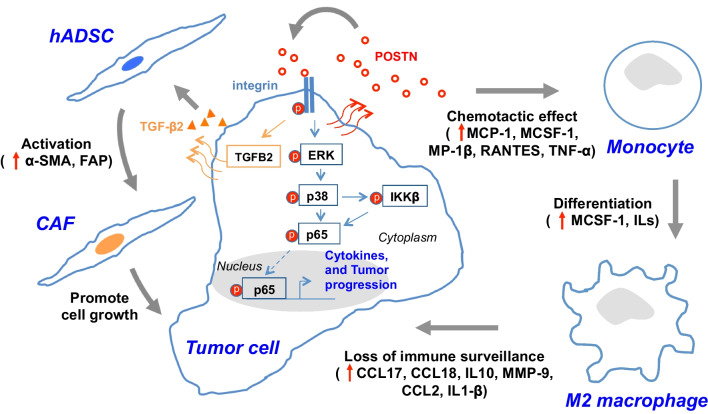
Model for POSTN modulated ovarian cancer malignancy. The diagram illustrates POSTN-mediated signaling axis to induce ERK-p38 and IKKβ-mediated NF-κB activation. This leads to cytokines/chemokines production, as well as monocyte mobilization and differentiation to enrich M2 macrophages and also induces TGF-β2 expression to activate CAFs in the tumor microenvironment

Our study elucidated the POSTN-mediated interplay between ovarian cancer cells and stroma to promote tumor growth and metastasis. Unlike most of the published reports showing that POSTN is produced by tumor stroma cells such as CAFs to exert its effect [44], we have unveiled an autocrine effect of POSTN triggering integrin dependent activation of IKKβ-mediated NF-κB and TGF-β2 in ovarian cancer cells. This leads to cytokine/chemokine production from cancer cells to facilitate monocyte infiltration and differentiation to enrich M2 macrophages in the tumor microenvironment, at the same time, POSTN also mediates TGF-β2 expression to promote CAF activation (Fig. 7). TGF-β1 and TGF-β2 share ~ 70–80% sequence identity and signaling through the same receptor. Considering there could be released from different cell sources in ovarian TME [31, 45] to interact with the same receptor, it is likely that there may exist a synergistic effect of TGF-β(s) from different cell sources on activation of CAFs. Both M2 macrophages and CAFs are known to play important roles in facilitating tumor progression and metastasis [12, 46–48]. Our findings are consistent with the report that in a xenograft model POSTN secreted by glioblastoma stem cells was shown to help to recruit tumor-supportive TAMs via integrin α_v_β_3_ signaling [30]. In addition, upregulation of the NF-κB pathway not only stimulates tumor promoting inflammatory responses, but also activates Snail and β-catenin [49, 50] resulting in epithelial-mesenchymal transition and invasion [50]. Previous studies showed that overexpression of POSTN predicted a poor prognosis in non-small cell lung cancer and colorectal cancer and suggested that POSTN could be a regulator of inflammation in the tumor microenvironment and a potential therapeutic target in lung and colorectal cancers [51, 52]. Here we show that POSTN plays an important role in ovarian tumor metastasis via cancer cells autocrine effect to enrich M2 macrophages and CAFs. Elevated expression of POSTN is associated with advanced stages of ovarian tumors and poor survival of patients.

It has been known that TAMs may be derived from circulating monocytes or resident macrophages in tumor microenvironment [53]. Growth factors, such as colony-stimulating factor-1 (CSF-1; M-CSF) and interleukins (ILs), can regulate the mononuclear phagocytic cells for their development and functions [54, 55]. Our study revealed an alternative POSTN/integrin/ERK/NF-κB pathway to promote recruitment of monocytes and differentiation into M2 macrophage-like TAMs in ovarian tumor microenvironment, which could contribute to compromising the tumor immune surveillance through M2 macrophage-associated immune suppressive cytokines (Figs. 5, 7). It is worthwhile noting that a large panel of cytokines including MCP-1, M-CSF (CSF-1), IL-3, IL-10, IL-12, IL-16, MIP-1β, RANTES, TNF-α and TGF-β2 were upregulated resulting from autocrine effect in POSTN-overexpressing SKOV3 cells leading to potent enrichment of TAMs in the tumor microenvironment (Fig. 7). Although undoubtedly, activation of ERK and NF-κB plays a key role in the induction of those cytokines, we cannot exclude the potential contribution from other signaling components, such as FAK and Src, downstream of the integrin signaling.

In addition to TAMs, many studies have shown the tumor promoting effect of CAFs [38, 56]. Tumor stroma CAF-derived POSTN has been shown to promote head and neck cancer stemness by activating protein tyrosine kinase 7-Wnt/β-catenin signaling [32]. In ovarian cancer, CAFs were shown to be responsible for the deposition of POSTN, which was able to decrease cisplatin-induced apoptosis potentially through the PI3K/AKT signaling pathway [20, 57]. In addition, the interplay between cancer cells and peritumoral stromal cells was shown to cause carboplatin and paclitaxel chemoresistance due to high POSTN expression [19]. Our results are also in agreement with a previous study showing that ascites from ovarian cancer patients contained a high level of POSTN, which functioned as a ligand for α_v_β_3_ and α_v_β_5_ integrins, thereby activating their signaling and promoting the adhesion and migration ability of ovarian cancer cells [17]. However neither of the above two reports [17, 19] elucidated the underlying mechanism accounting for POSTN-mediated chemoresistance or enhanced metastasis of ovarian cancer cells. Our study shows that autocrine effect of cancer cells-released POSTN can trigger NF-κB and TGF-β2 pathways to enrich M2 macrophages and CAFs in ovarian tumor microenvironment.

## Conclusion

Overall, the present study unveiled the POSTN-mediated interplay between ovarian cancer cells and stroma including monocytes and CAFs, and elucidated the underlying mechanism involved in cancer progression and metastasis. Our study suggests that POSTN not only can serve as a prognosis marker, but also a therapeutic target for ovarian cancer. Development of a POSTN small molecular inhibitor interfering with its binding to integrin or a blocking monoclonal antibody could be worthwhile as new ovarian cancer therapeutics.

## Supplementary Information

**Additional file 1**: **Fig. S1**. The expression of POSTN is elevated in the late stage ovarian cancer patients*, *and POSTN regulates migratory, invasive, colony forming and cell adhering abilities of ovarian cancer cells. **Fig. S2**. POSTN promotes NF-κB and its related signaling molecules, is colocalized with integrin β3 and integrin β5 and regulates ovarian cancer cell migration and invasion. **Fig. S3**. *POSTN*-silencing reduces ovarian cancer malignancy in vivo*. ***Fig. S4**. Direct effect of POSTN on THP-1 migration and differentiation. **Fig. S5**. *POSTN* expression is associated with increasing abundance of CAF. **Table S1**. Reagents used in this study. **Table S2**. siRNA and shRNA clones used in this study. **Table S3**. Antibodies used in this study. **Table S4**. qPCR primers used in this study. **Table S5**. Summary of TissueScan cohort (#HORT102, OriGene). **Table S6**. Summary of ovarian cancer patients from CMUH (IRB#: CMUH 107-REC1-095).

## Data Availability

The datasets used for the current study are available from the corresponding author on reasonable request.

## Abbreviations

POSTN: Periostin
EOC: Epithelial ovarian cancer
TAM: Tumor-associated macrophage
CAF: Cancer-associated fibroblast
ECM: Extracellular matrix
NF-κB: Nuclear factor kappa-light-chain-enhancer of activated B cells
TGF-β: Transforming growth factor beta
ERK: Extracellular signal-regulated kinases
MIP-1β: Macrophage inflammatory protein-1 beta
MCP-1: Monocyte chemoattractant protein-1
TNFα: Tumor necrosis factor α
RANTES: Regulated upon activation, normal T cell expressed and presumably secreted
α-SMA: Alpha-smooth muscle actin
FAP: Fibroblast activation protein
OSE: Ovarian surface epithelium
IKKα: IκB kinase α
IKKβ: IκB kinase β
GI: Gastrointestinal
CD206: Cluster of differentiation 206
CD80: Cluster of differentiation 80
CD68: Cluster of differentiation 68
CCL2: C–C motif chemokine ligand 2
CCL17: C–C motif chemokine ligand 17
CCL18: C–C motif chemokine ligand 18
IL-10: Interleukin 10
MMP-9: Matrix metalloproteinase 9
CCL11: C–C motif chemokine ligand 11
IL-12: Interleukin 12
IL-3: Interleukin 3
IL-16: Interleukin 16
MCP-1: Monocyte chemoattractant protein-1
MCSF: Macrophage colony-stimulating factor
TPCA-1: 2-[(Aminocarbonyl)amino]-5-(4-fluorophenyl)-3-thiophenecarboxamide
hADSCs: Human adipose derived stromal cells
qRT-PCR: Quantitative real-time reverse transcription polymerase chain reaction
FAK: Focal adhesion kinase
Src: Proto-oncogene tyrosine-protein kinase Src
PTK7: Protein tyrosine kinase 7
PI3K: Phosphoinositide 3-kinase
