# Correction: Periostin promotes ovarian cancer metastasis by enhancing M2 macrophages and cancer-associated fibroblasts via integrin-mediated NF-κB and TGF-β2 signaling

**DOI:** 10.1186/s12929-023-00948-w

**Published:** 2023-07-12

**Authors:** Sheng-Chieh Lin, Yi-Chu Liao, Po-Ming Chen, Ya-Yu Yang, Yi-Hsiang Wang, Shiao-Lin Tung, Chi-Mu Chuang, Yu-Wen Sung, Te-Hsuan Jang, Shuang-En Chuang, Lu-Hai Wang

**Affiliations:** 1 Graduate Institute of Integrated Medicine and Chinese Medicine Research Center, China Medical University, No. 91, Hsueh Shih Road, Taichung, 40402 Taiwan; 2 Graduate Institute of Biomedical Sciences, China Medical University, Taichung, Taiwan; 3 Institute of Molecular and Genomic Medicine, National Health Research Institutes, Miaoli, Taiwan; 4 Research Assistant Center, Show Chwan Memorial Hospital, Changhua, Taiwan; 5 National Institute of Cancer Research, National Health Research Institutes, Miaoli, Taiwan; 6 Institute of Molecular Medicine, National Tsing Hua University, Hsinchu, Taiwan; 7 Department of Hematology and Oncology, Ton-Yen General Hospital, Hsinchu, Taiwan; 8 Department of Nursing, Hsin Sheng Junior College of Medical Care and Management, Taoyuan, Taiwan; 9 Department of Obstetrics and Gynecology, Taipei Veterans General Hospital, Taipei, Taiwan; 10 School of Medicine, National Yang-Ming University, Taipei, Taiwan; 11 Department of Obstetrics and Gynecology, China Medical University Hospital, Taichung, Taiwan

**Correction: Journal of Biomedical Science (2022) 29:109** **https://doi.org/10.1186/s12929-022-00888-x**

Following publication of the original article [1], it was noticed that the Funding information need to be corrected, the correct Funding section is shown as below:

This work was supported by the following sources: Ministry of Science and Technology (MOST107-2320-B-039-059-MY3 and 109-2320-B-039-060); Ministry of Health and Welfare (MOHW110-TDU-B-2-11-010001), Executive Yuan, Taiwan; and China Medical University (CMU107-TU-11, CMU110-Z-06, and CMRC-CENTER-0), Taichung, Taiwan.

The original paper has been updated.
