# Supplementary material for: Periostin promotes ovarian cancer metastasis by enhancing M2 macrophages and cancer-associated fibroblasts via integrin-mediated NF-κB and TGF-β2 signaling
Source: J Biomed Sci. 2022 Dec 22;29:109. doi: 10.1186/s12929-022-00888-x (PMC9784270; doi:10.1186/s12929-022-00888-x)
Supplement: Supplementary file 1 — Additional file 1: Fig. S1. The expression of POSTN is elevated in the late stage ovarian cancer patients, and POSTN regulates migratory, invasive, colony forming and cell adhering abilities of ovarian cancer cells. Fig. S2. POSTN promotes NF-κB and its related signaling molecules, is colocalized with integrin β3 and integrin β5 and regulates ovarian cancer cell migration and invasion. Fig. S3. POSTN-silencing reduces ovarian cancer malignancy in vivo. Fig. S4. Direct effect of POSTN on THP-1 migration and differentiation. Fig. S5. POSTN expression is associated with increasing abundance of CAF. Table S1. Reagents used in this study. Table S2. siRNA and shRNA clones used in this study. Table S3. Antibodies used in this study. Table S4. qPCR primers used in this study. Table S5. Summary of TissueScan cohort (#HORT102, OriGene). Table S6. Summary of ovarian cancer patients from CMUH (IRB#: CMUH 107-REC1-095). [file 12929_2022_888_MOESM1_ESM.docx]

**Additional file 1**

**Periostin Promotes Ovarian Cancer Metastasis by Enhancing M2 Macrophages and Cancer-associated Fibroblasts via Integrin-mediated NF-κB and TGF-β2 Signalling**

Sheng-Chieh Lin, Yi-Chu Liao, Po-Ming Chen, Ya-Yu Yang, Yi- Hsiang Wang, Shiao-Lin Tung, Chi-Mu Chuang, Yu-Wen Sung, Te-Hsuan Jang, Shuang-En Chuang and Lu-Hai Wang*

**This file includes:**

Additional Figures:

Fig. S1. The expression of POSTN is elevated in the late stage ovarian cancer patients*,* and POSTN regulates migratory, invasive, colony forming and cell adhering abilities of ovarian cancer cells.

Fig. S2. POSTN promotes NF-κB and its related signaling molecules, is colocalized with integrin β3 and integrin β5 and regulates ovarian cancer cell migration and invasion.

Fig. S3. *POSTN*-silencing reduces ovarian cancer malignancy *in vivo.*

Fig. S4. Direct effect of POSTN on THP-1 migration and differentiation.

Fig. S5. *POSTN* expression is associated with increasing abundance of CAF.

Additional Tables:

Table S1. Reagents used in this study

Table S2. siRNA and shRNA clones used in this study

Table S3. Antibodies used in this study

Table S4. qPCR primers used in this study

Table S5. Summary of TissueScan cohort (#HORT102, OriGene)

Table S6. Summary of ovarian cancer patients from CMUH (IRB#: CMUH 107-REC1-095)

**Figures:**

**POSTN**

**Actin**

**SKOV-I6**

**A2780**

**F1120**

**OVS1**


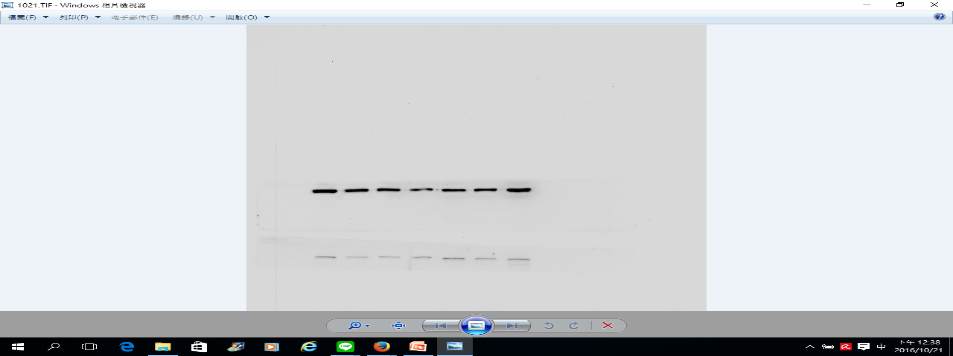

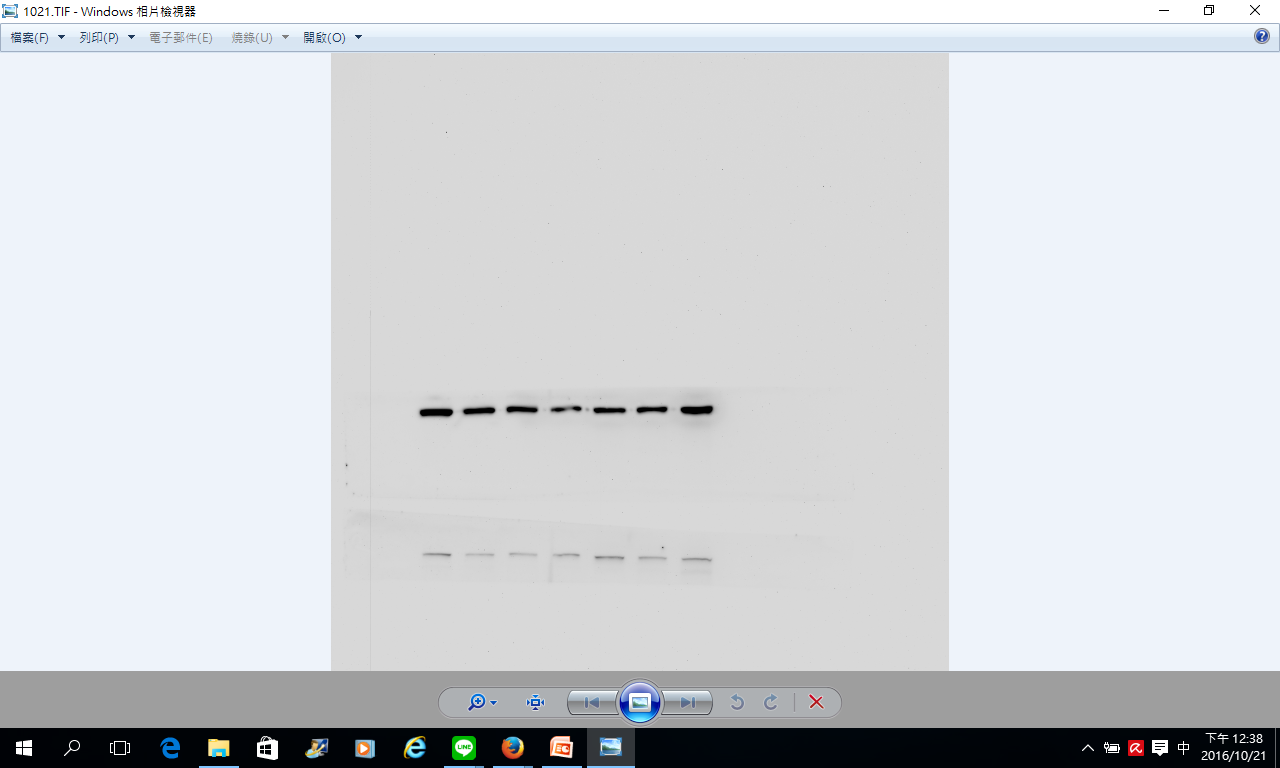

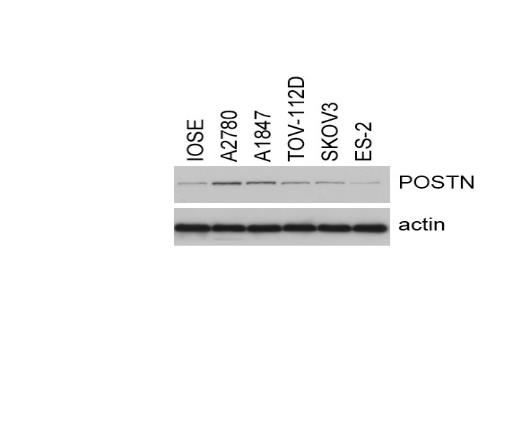


**IOSE**

**A2780**

**A1847**

**TOV-112D**

**SKOV3**

**ES-2**


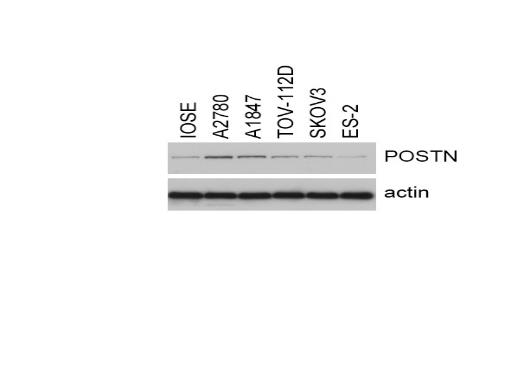


**A**

**
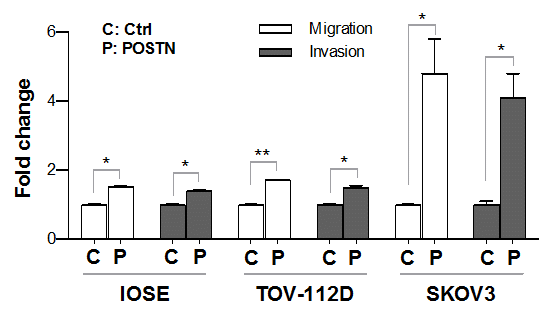
**

**B**


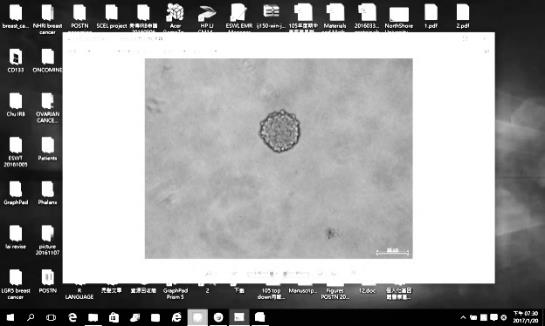


**50 μm**


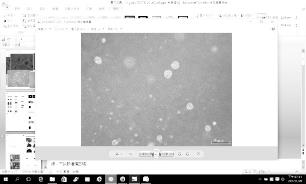

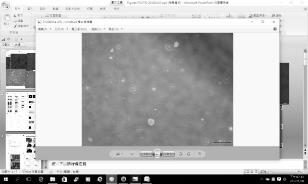


**SC**

**shPOSTN#1**

**shPOSTN#2**

**OVS1**


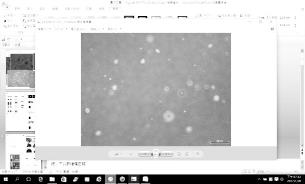

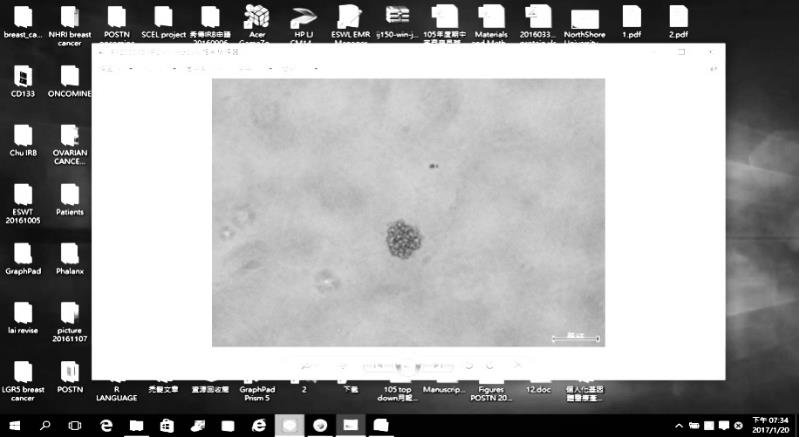


**50 μm**

**50 μm**

**50 μm**


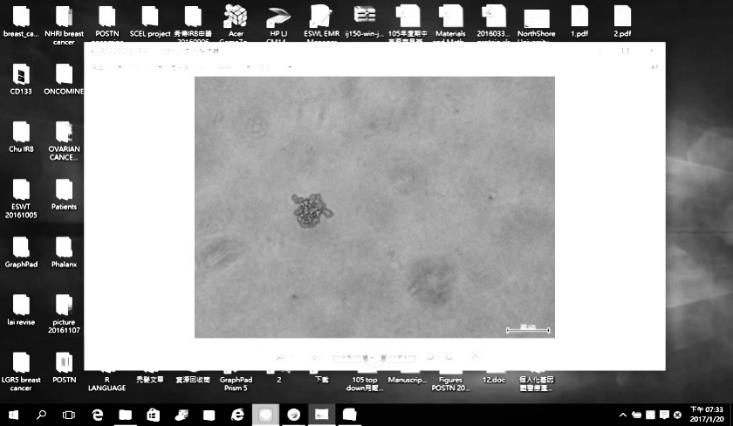


**50 μm**

**50 μm**

**OVS1**

**SC**

**shPOSTN#1**

**shPOSTN#2**

**POSTN**

**Actin**


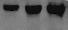

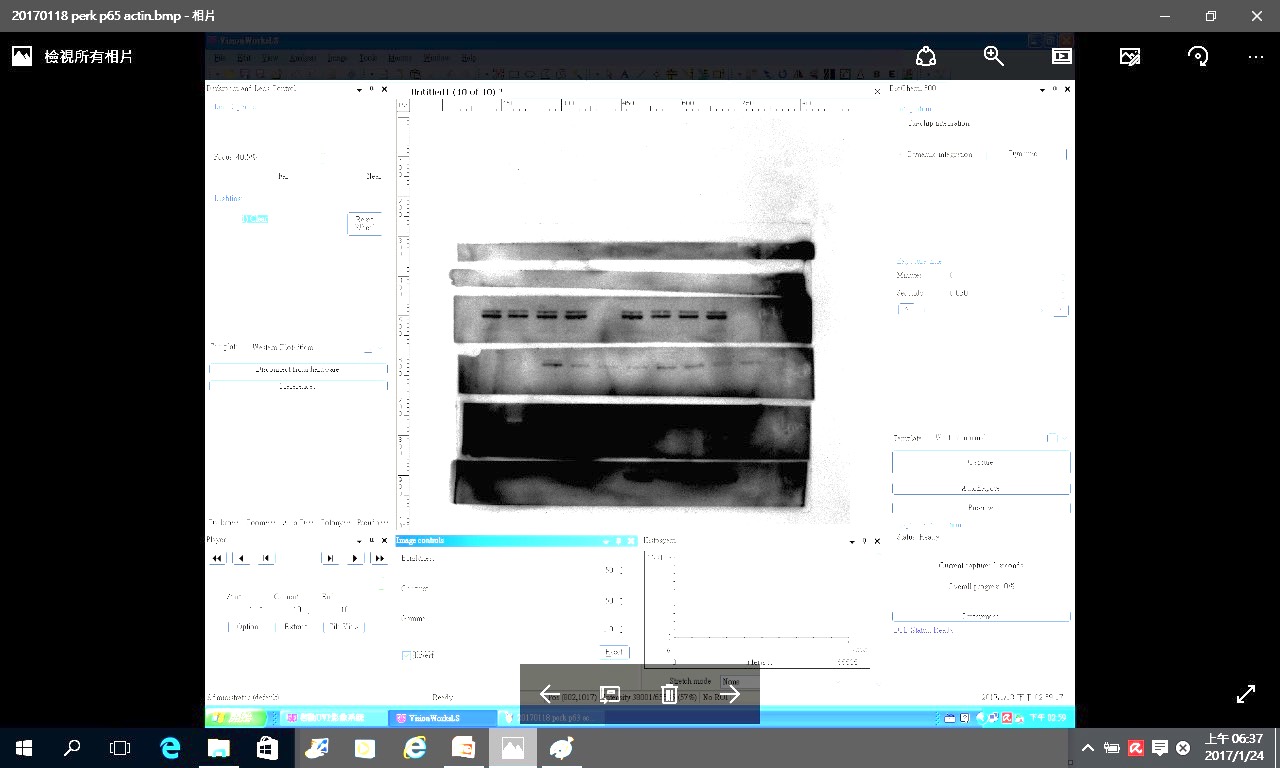


**D**

**C**


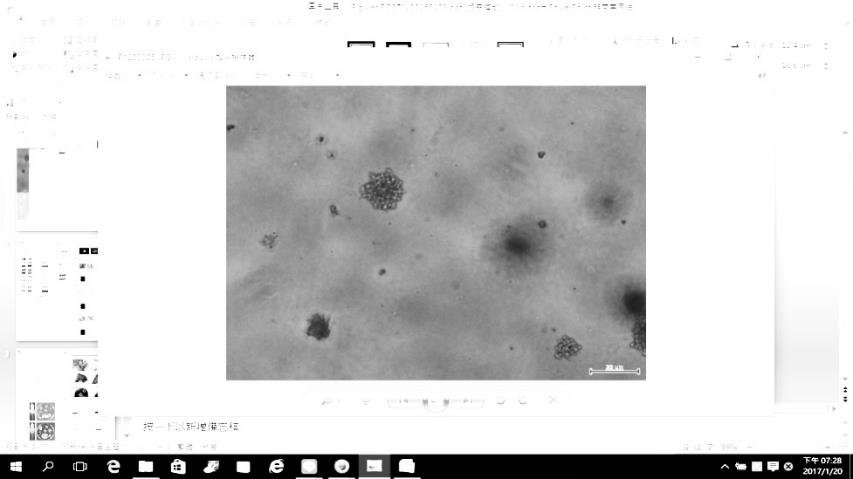

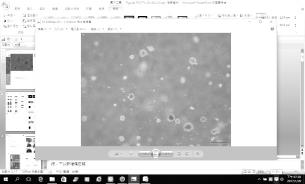


**Ctrl**

**POSTN**

**TOV-112D**


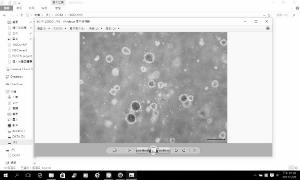

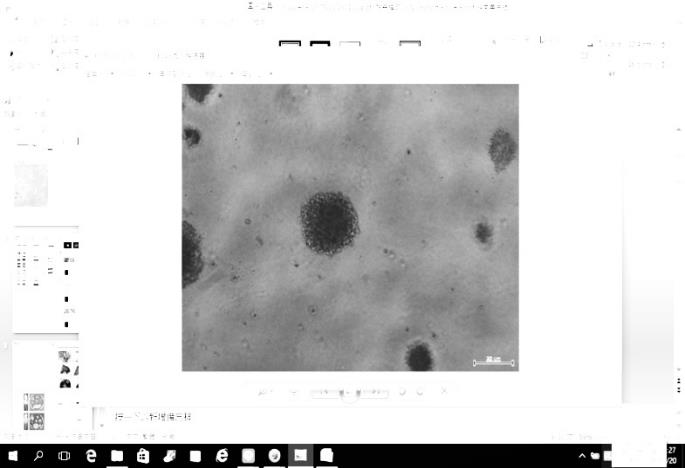


**50 μm**

**50 μm**

**50 μm**

**50 μm**

**TOV-112D**

**Ctrl**

**POSTN**

**POSTN**

**Actin**


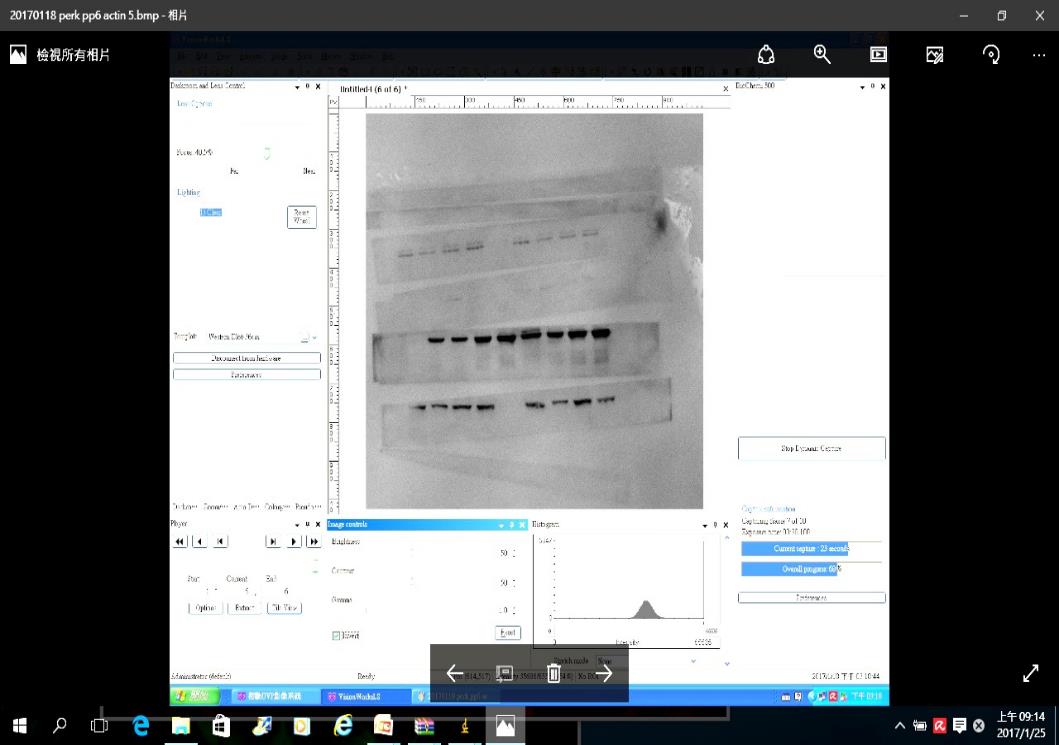

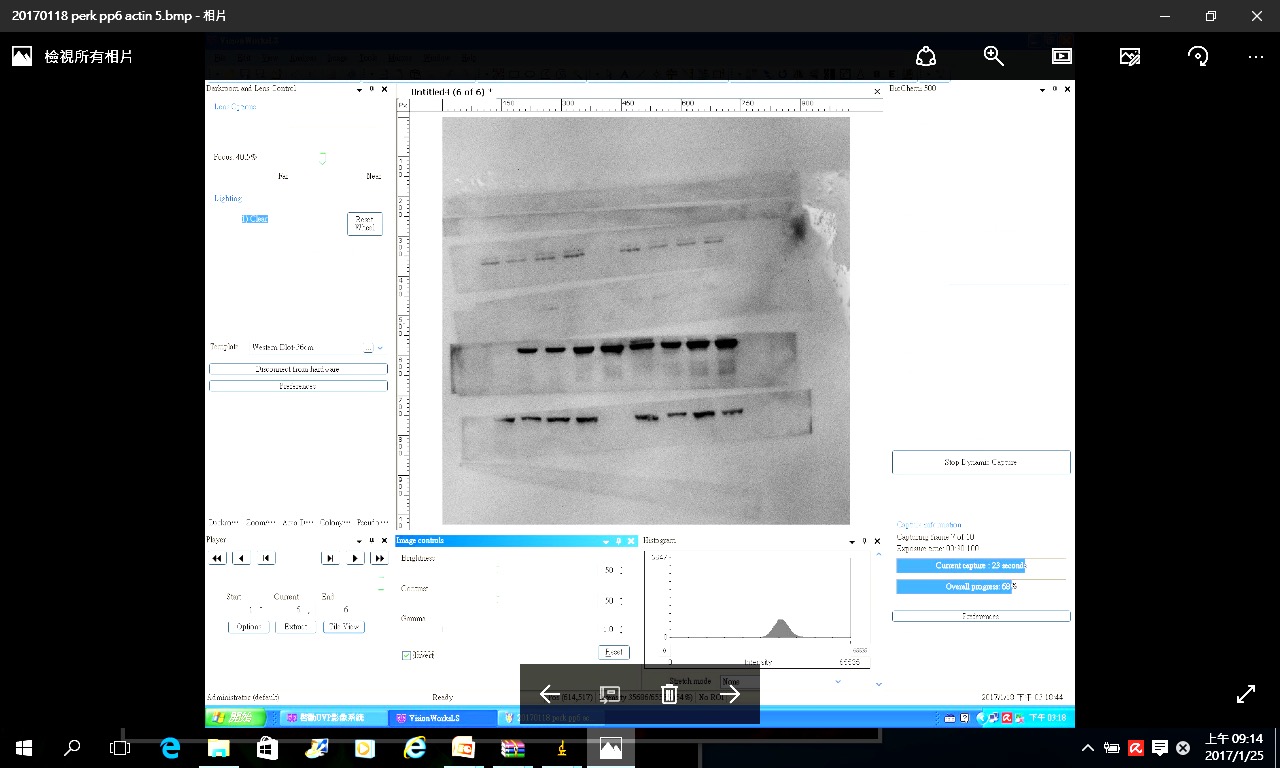


**C**

**SKOV-I6**

**E**

**F**


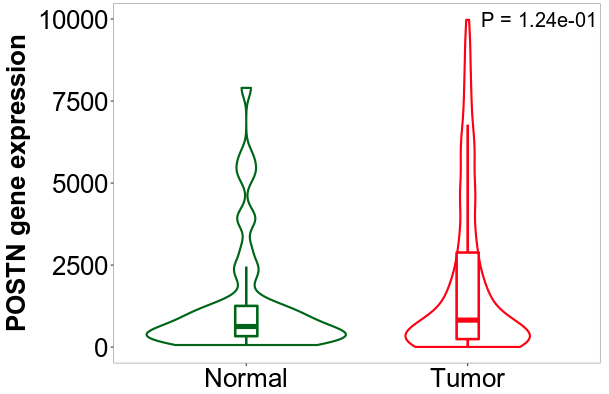


**H**

**G**

**Additional Fig. S1. The expression of POSTN is elevated in the late stage ovarian cancer patients*,* and POSTN regulates migratory, invasive, colony forming and cell adhering abilities of ovarian cancer cells.** **A** Endogenous POSTN expression of a panel of human ovarian cancer cell lines was evaluated by Western blotting. **B** Cell migration and invasion assay of IOSE, TOV-112D and SKOV3 cells with ectopic expression of POSTN. C: control. P: POSTN overexpression. **p* < 0.05, ***p* < 0.01. **C** and **D** Effect of knockdown or overexpression of POSTN in OVS1 and TOV-112D cells, respectively, on soft agar colony and sphere formation. 1×10^4^ cells were seeded per well in 6-well plates containing 0.4% low melting agar or sphere forming medium and cultured for 7 days. **E** Effect of POSTN on cell adhesion. Adhesion ability was assayed for SKOV3, SKOV-I6 transfected with control (N/S shRNA) or POSTN shRNA (shRNA#1 and #2). Data are presented as means ± SD. (****p* < 0.001). **F-H** RNA expression level of *POSTN* among different stages of ovarian cancer patients from online databases with larger ovarian cancer cohorts i.e. GENT2 (F), GEPIA2 (G) and TMNplot (H). * *p* < 0.05.

**A**

**B**

**B**


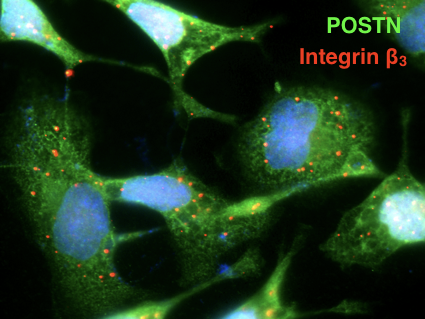


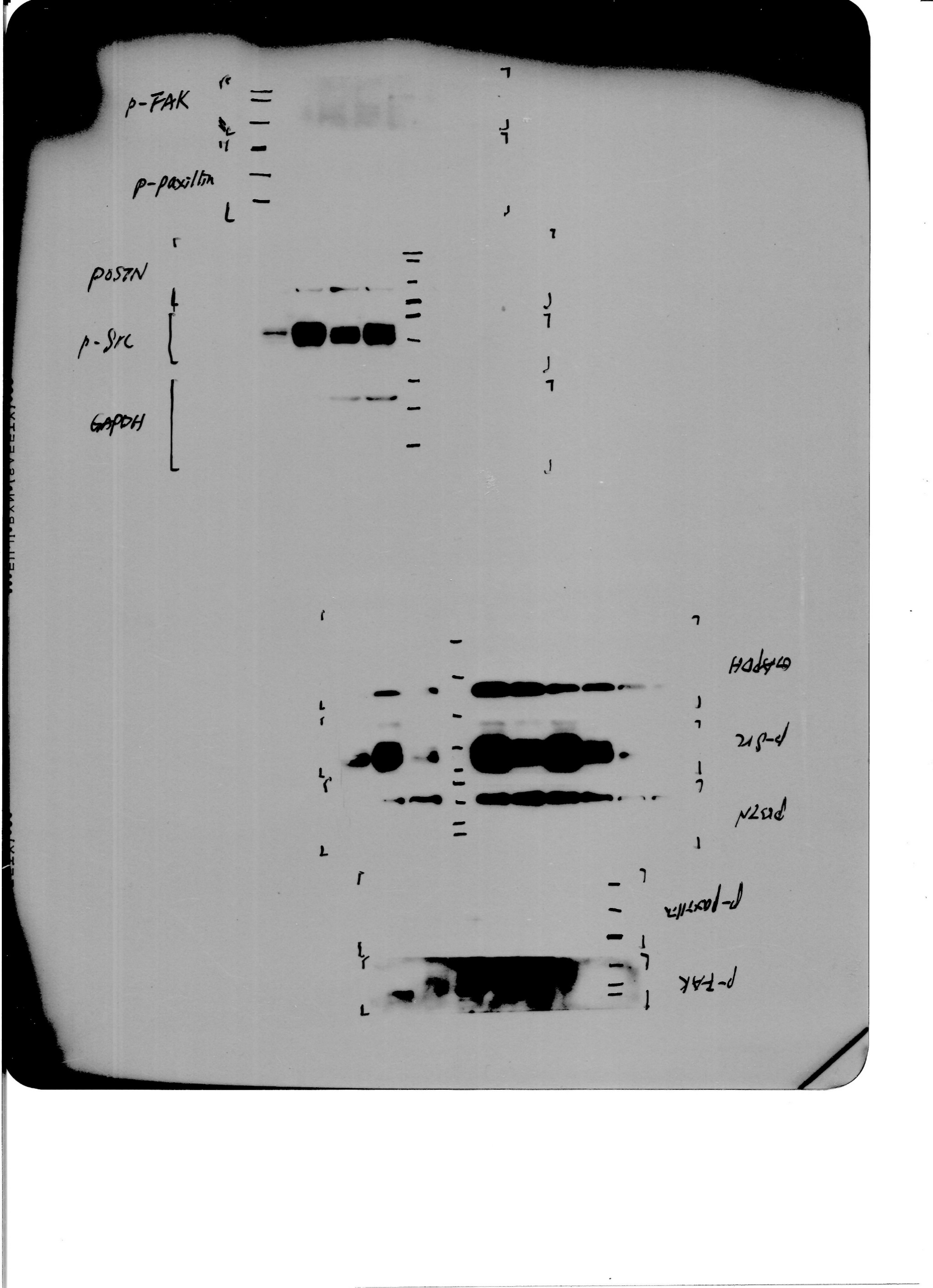

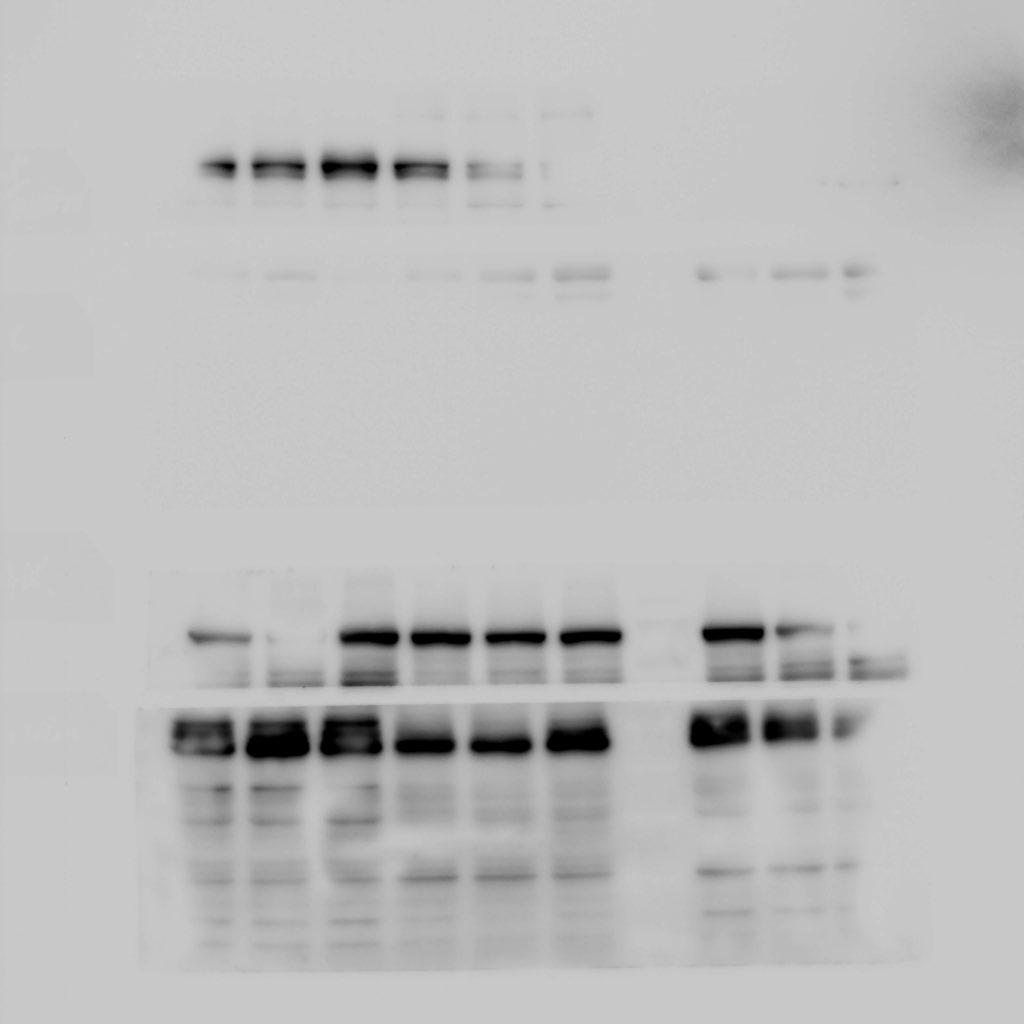

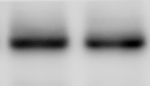

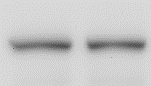

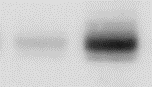

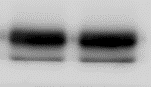

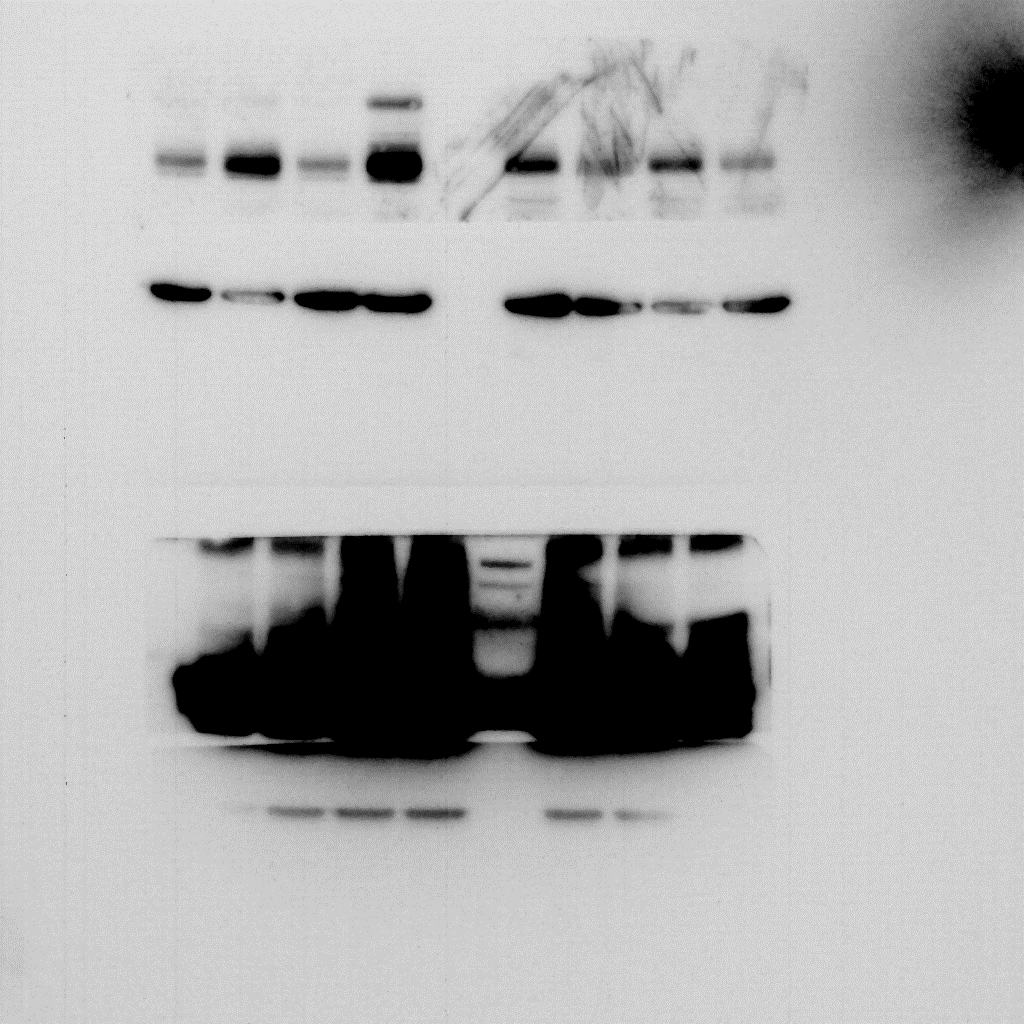

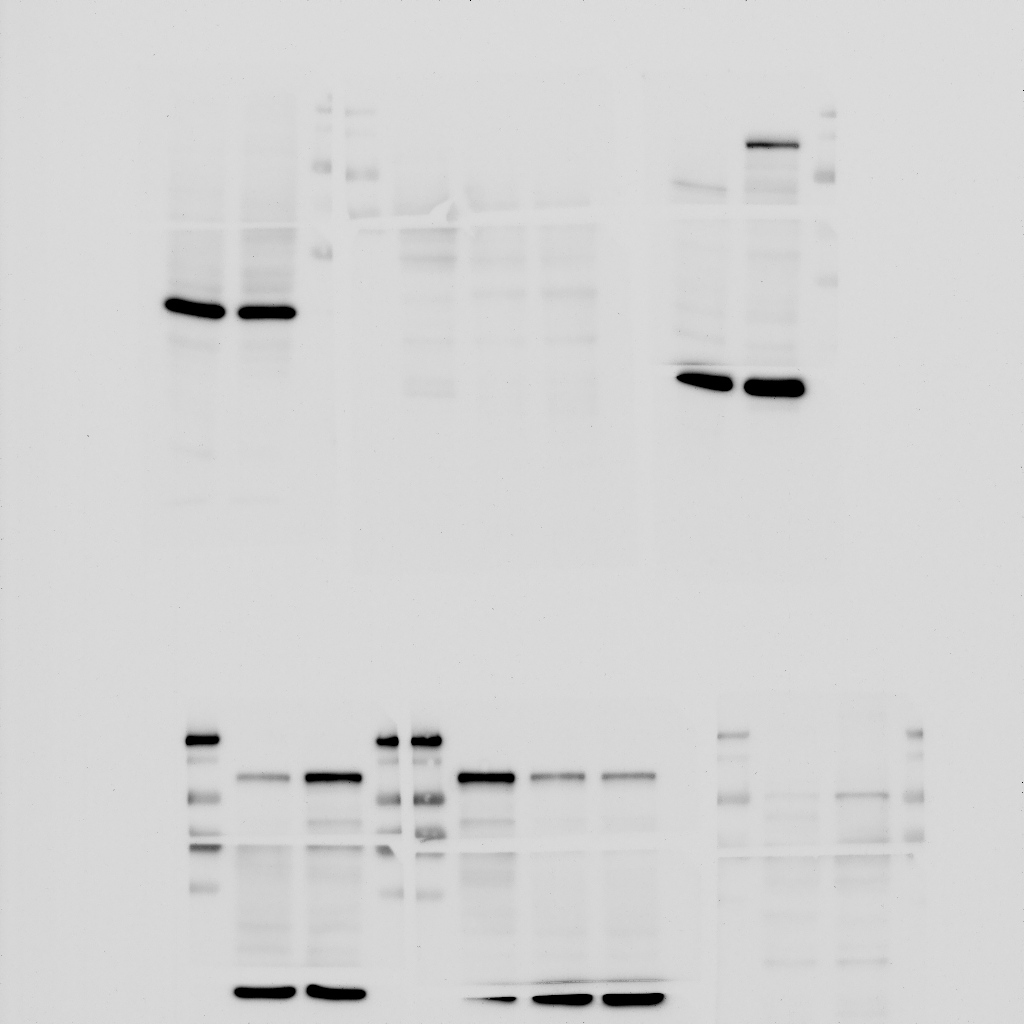

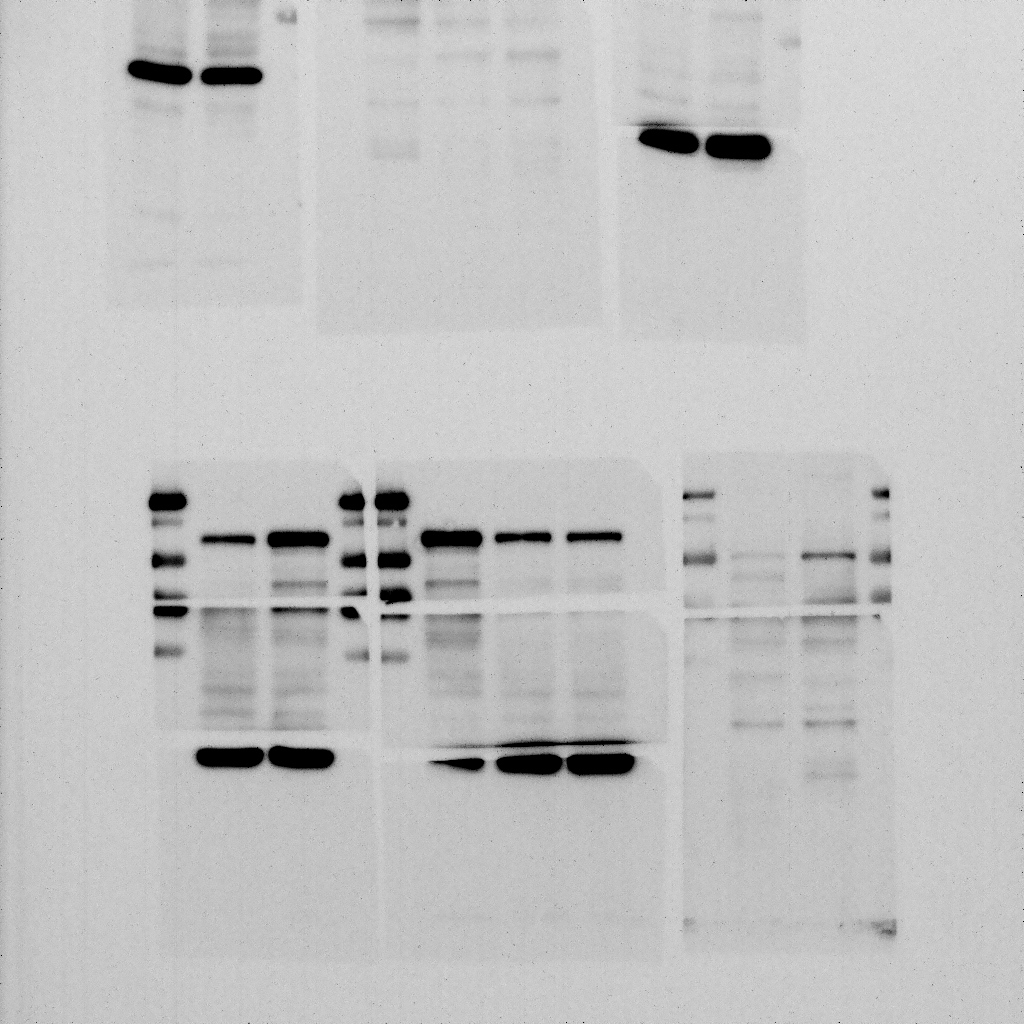

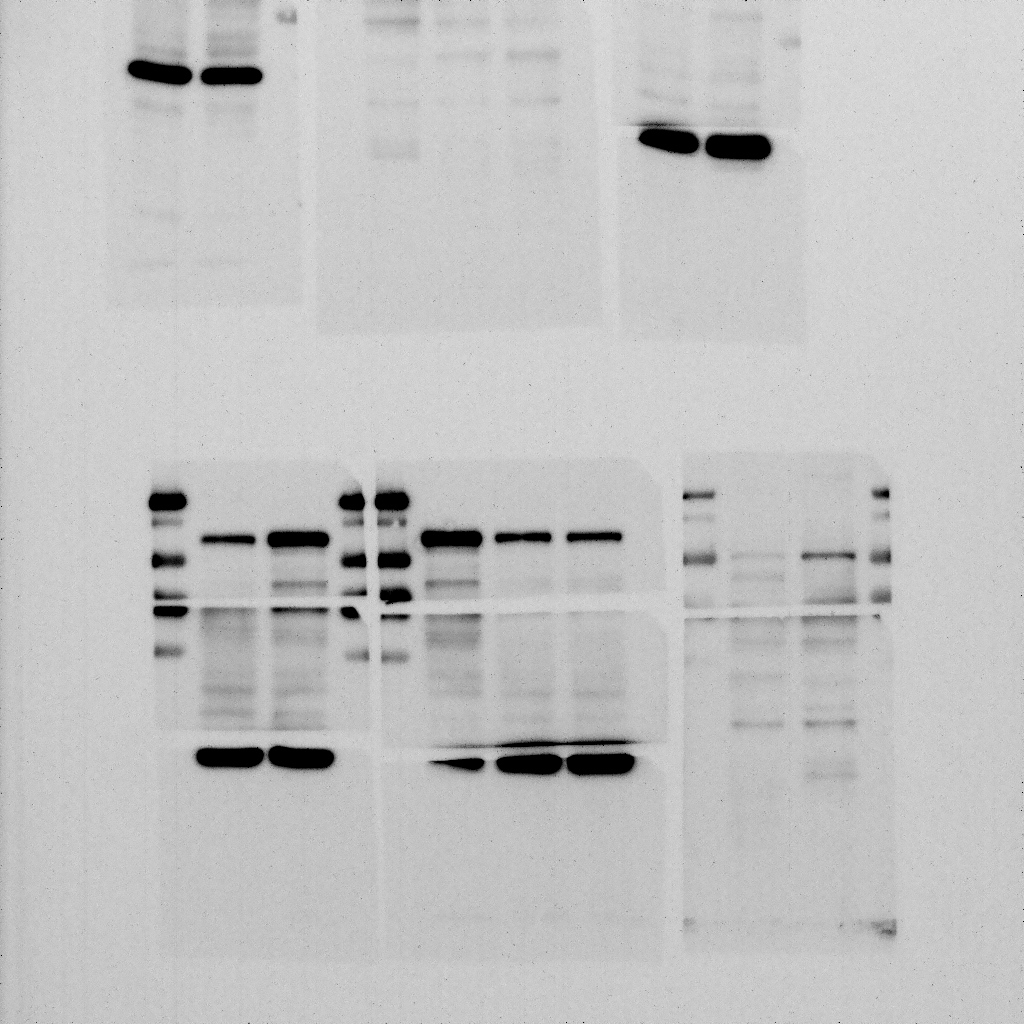

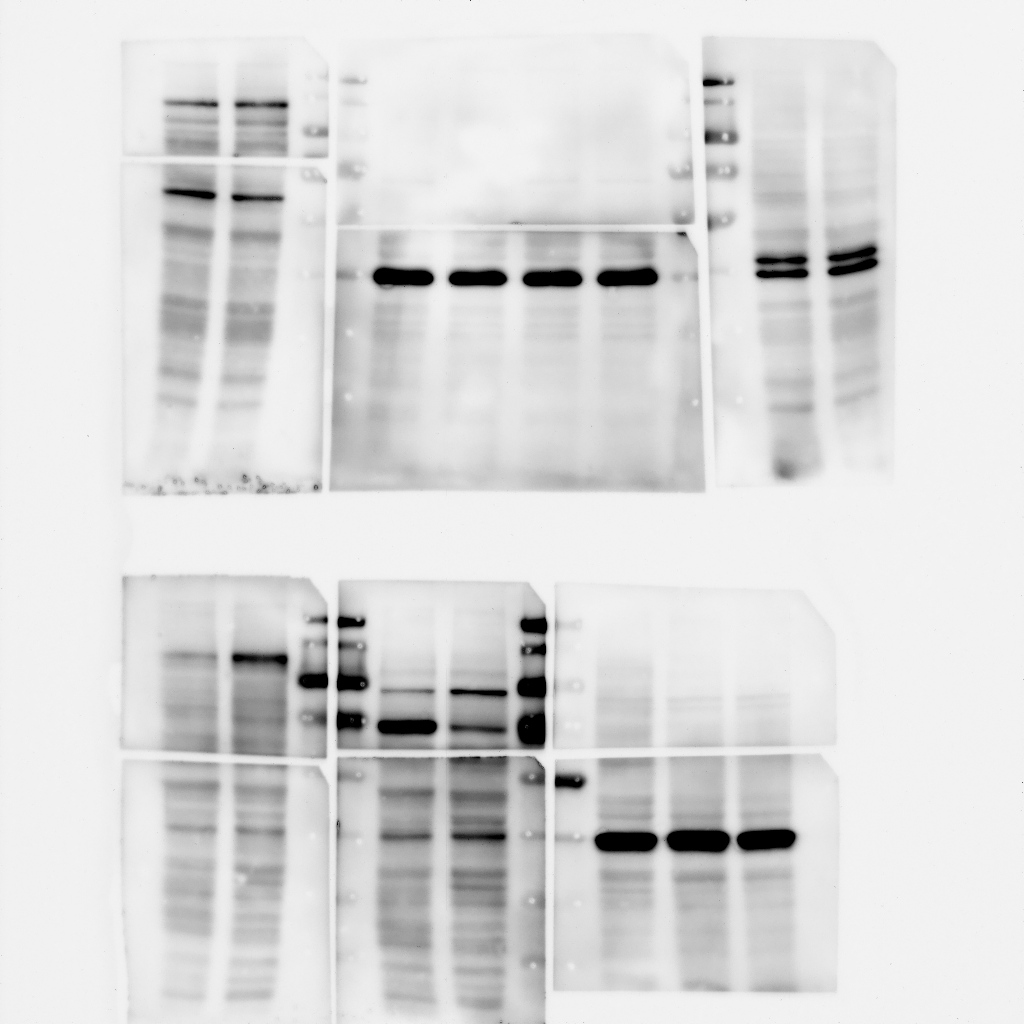

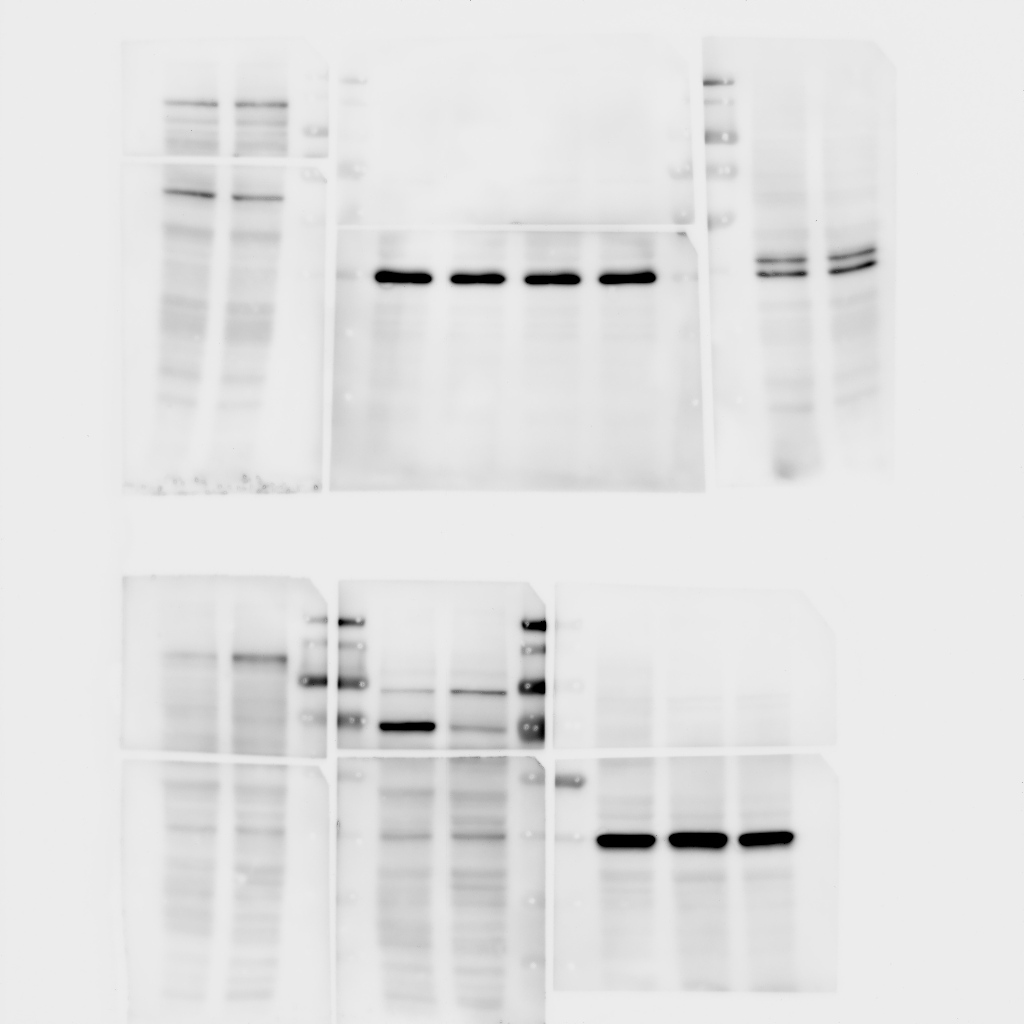

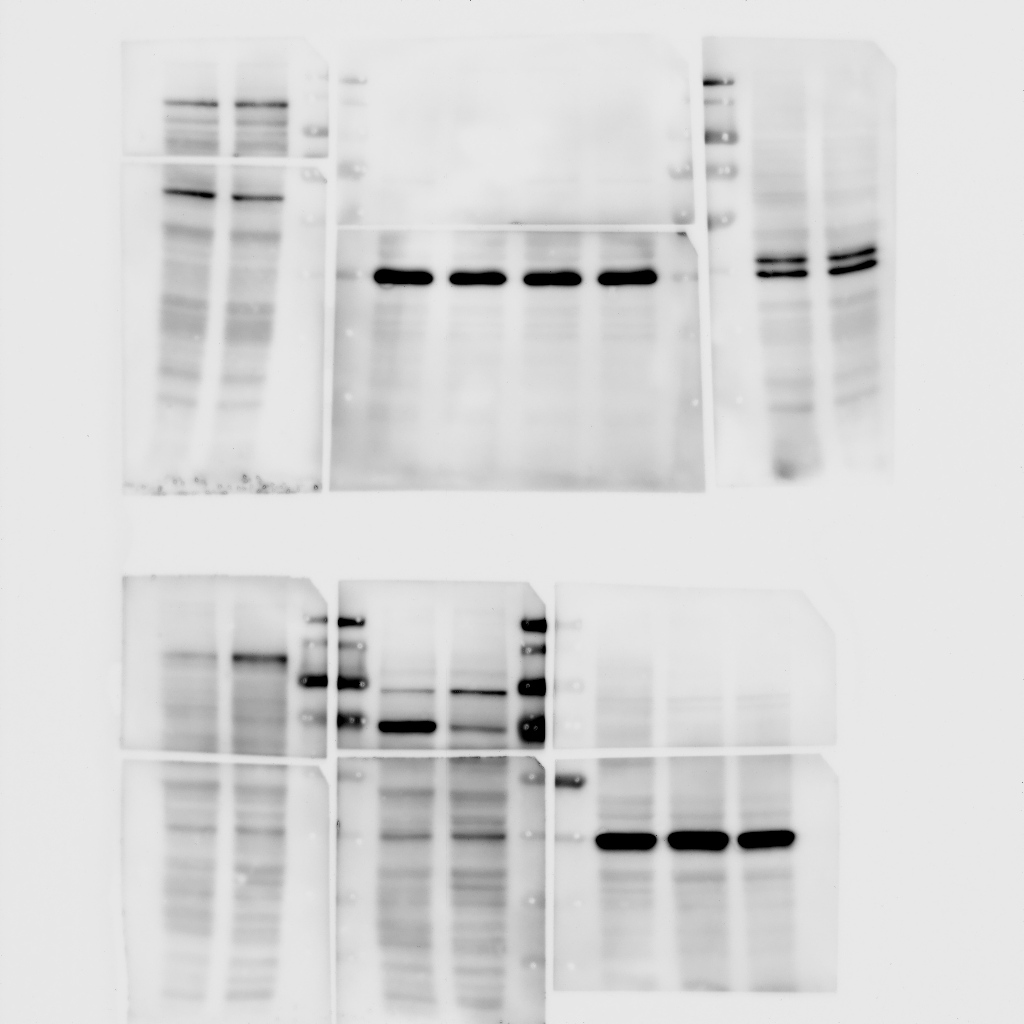

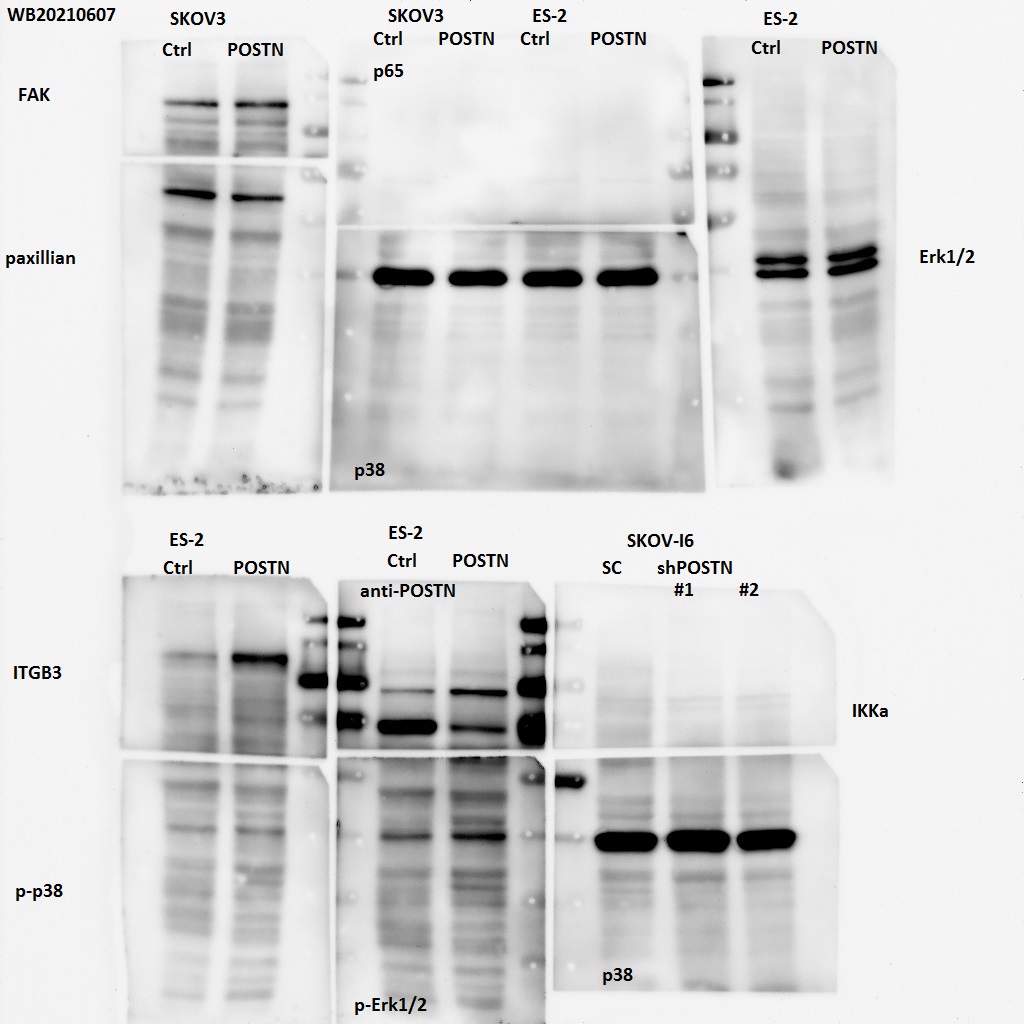

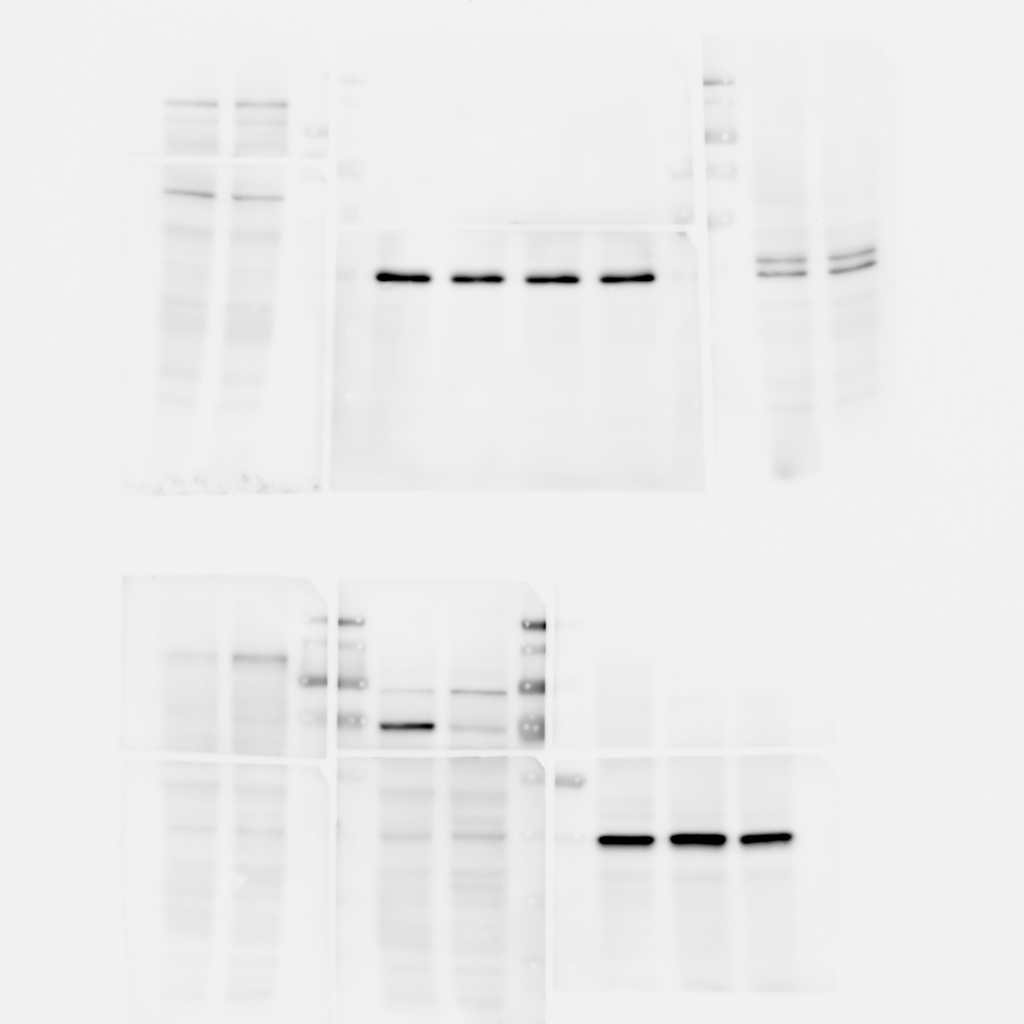

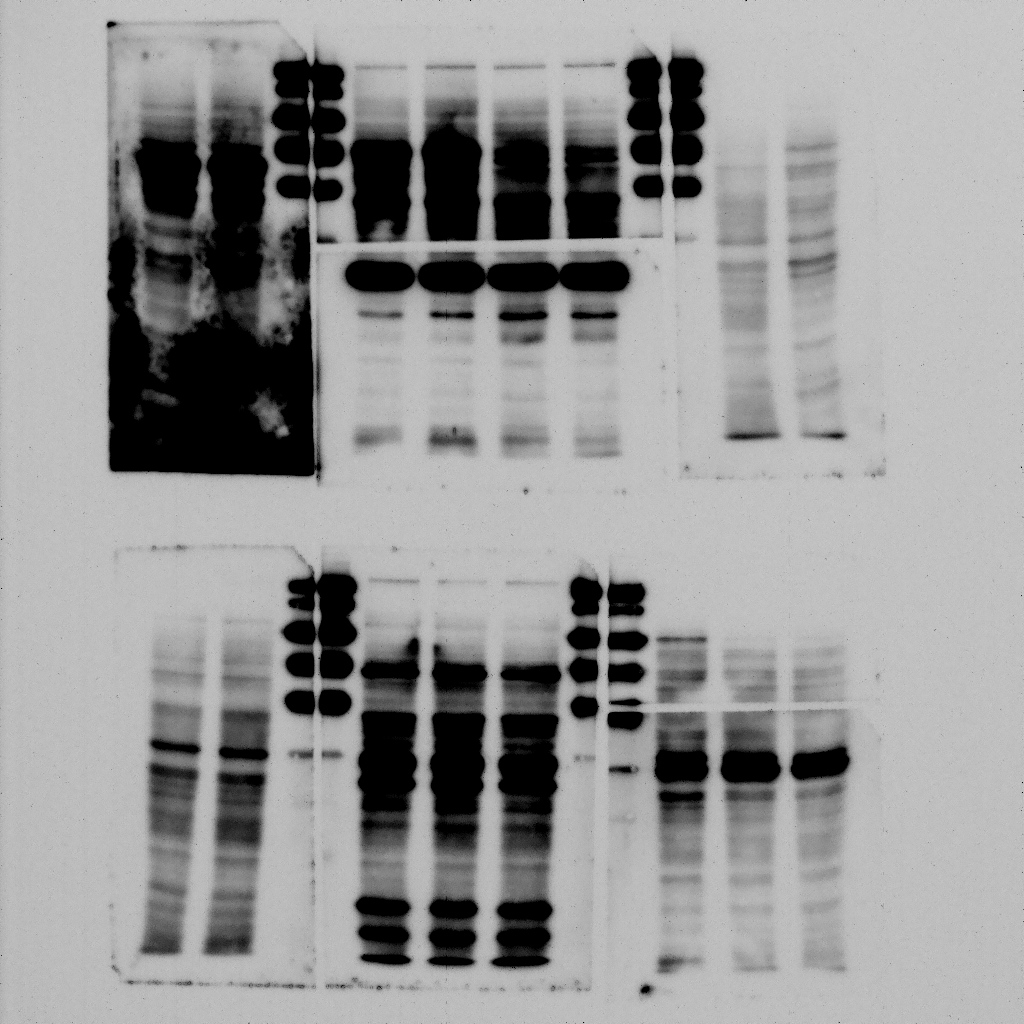

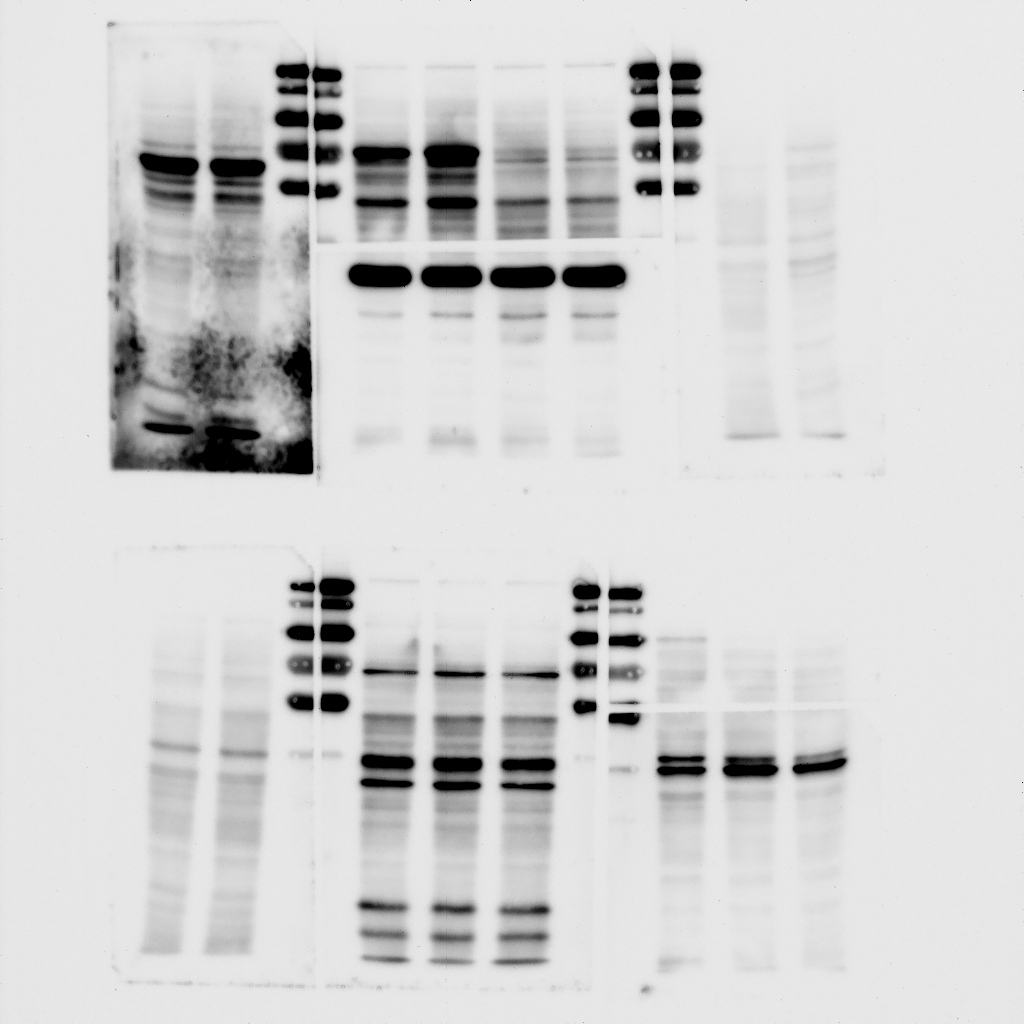

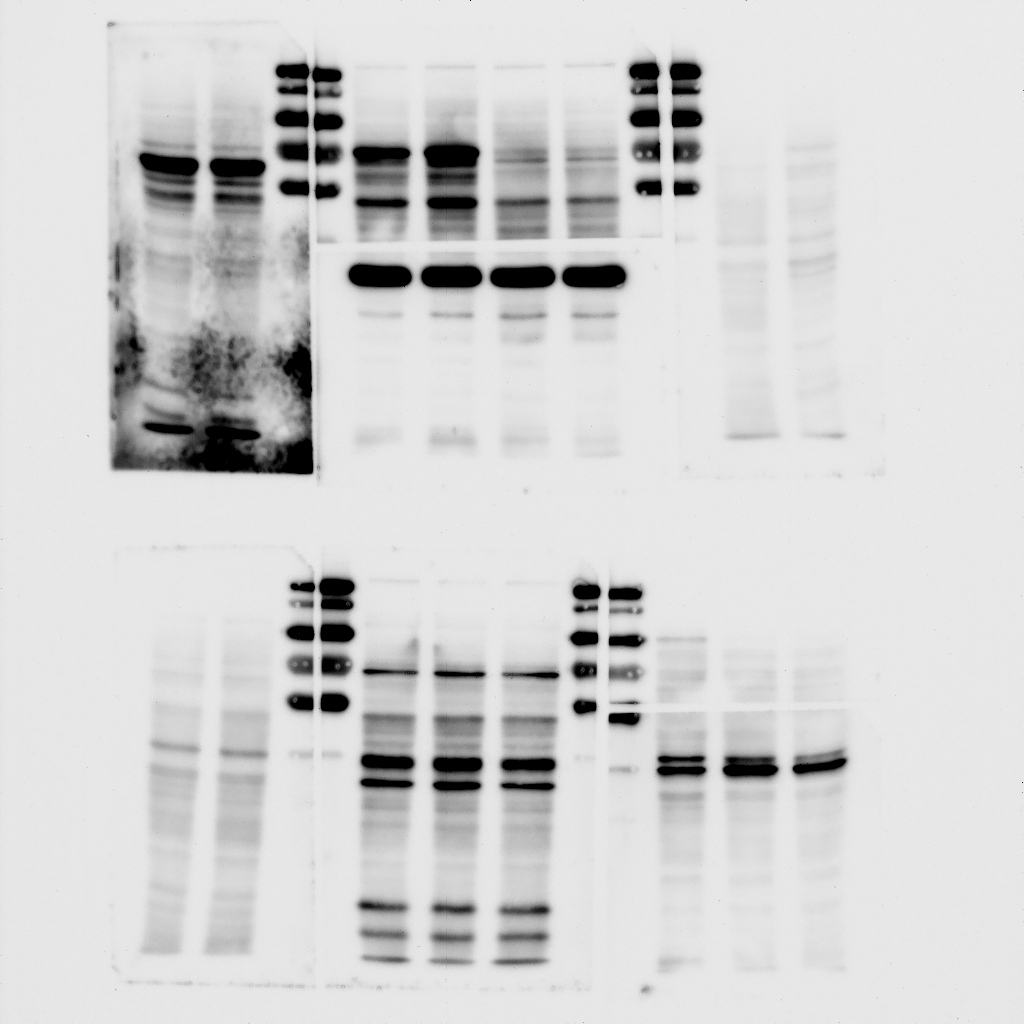


**ES-2**

**Ctrl**

**POSTN**

**Integrin β3**

**p-NFkB p65**

**p-p38**

**p-Erk**

**Erk**

**p38**

**NFkB p65**

**IKKα**

**IKKβ**

**POSTN**

**p-Paxillin (Y118)**

**p-FAK (Y861)**

**Integrin β5**

**FAK**

**p-Src (Y416)**

**Src**

**GAPDH**

**Paxillin**

**p65**

**Actin**

**IKKα**

**p-p38**

**p-p65**

**IKKβ**

**p38**


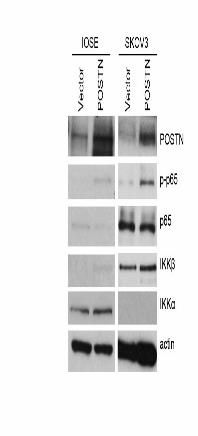

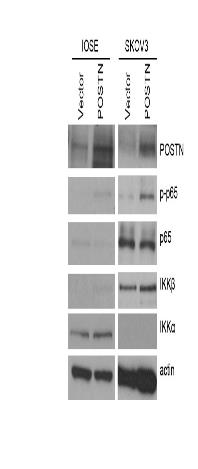

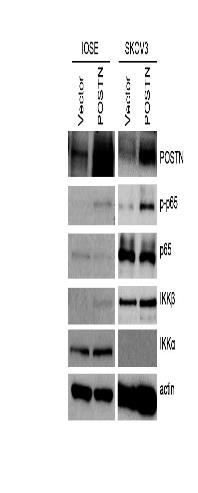

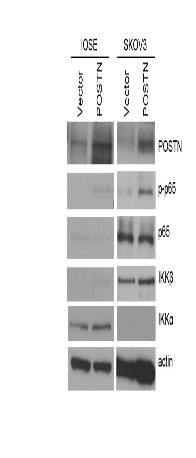

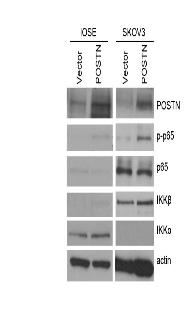

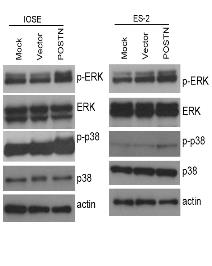

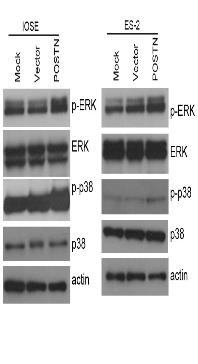

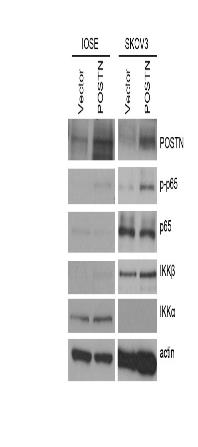


**Erk**

**p-Erk**

**POSTN**


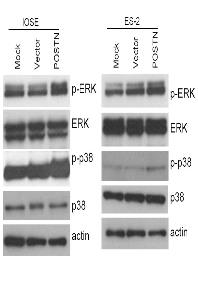

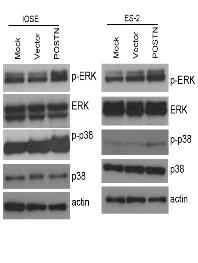


**IOSE**

**Ctrl**

**POSTN**

**CB**

**D**


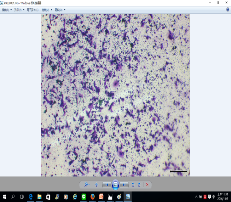

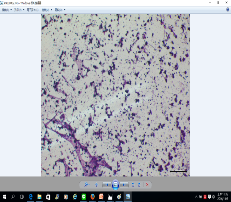

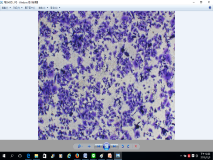

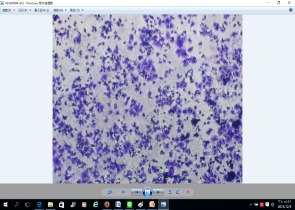


**E**

**OVS1**

**OVS1**

**IgG**

**α-POSTN**

**α-POSTN**

**(2 μg/mL)**

**(2 μg/mL)**

**IgG**

**Additional Fig. S2. POSTN promotes NF-κB and its related signaling molecules, is colocalized with integrin β3 and integrin β5 and regulates ovarian cancer cell migration and invasion. A** Correlation plot between *POSTN* and *NF-κB* (left), *POSTN* and *STAT1* (center), *POSTN* and *STAT3* (right) expression in ovarian cancer patients analyzed by GEPIA2. **B** Western blot analysis showing that overexpression of POSTN increased downstream signaling molecules of the NF-κB pathway. The expression of POSTN (**C, D,** green), integrin β3 (**C,** red) or integrin β5 (**D**, red) were distributed around the cell membrane boundary in SKOV-I6 cells. Nuclei were counterstained with DAPI (blue). Colocalized signals of POSTN and integrin β3 or integrin β5 were shown in yellow (white arrows). The scale bar represents 20 μm. **E** Migration and invasion assays of OVS1 cells treated with a POSTN specific monoclonal antibody (2 μg/mL) or control IgG. *** *p* < 0.001.

**
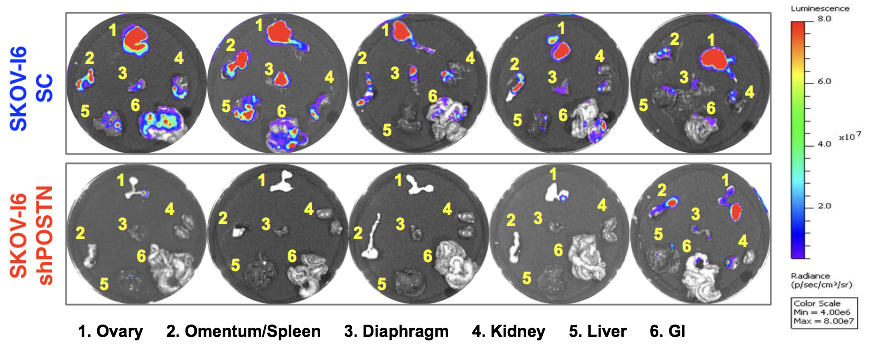
**

**Additional Fig. S3. *POSTN*-silencing reduces ovarian cancer metastasis *in vivo.*** Bioluminescent images of metastasized tumors in various organs of mice orthotopically implanted with *POSTN*-silenced SKOV3-I6 cells or scrambled control (SC) cells. Various organs of mice are as indicated.

**B**

**A**

**Additional Fig. S4. Direct effect of POSTN on THP-1 migration and differentiation.** THP-1 cells were pre-treated with PMA for 18 h followed by adding recombinant POSTN (rPOSTN) (200 ng/mL) for 48 h. **A** The migration ability of THP-1 cells increased upon rPOSTN treatment. **B** qPCR analysis was performed to detect CD68 (pan macrophage) (left), CD206 (M2 macrophage) (center) and CD80 (M1 macrophage) (right) expression in THP-1 cells with or without rPOSTN direct treatment. Results showed direct POSTN treatment promoted M2 macrophage differentiation. **p* < 0.05. (related to Fig. 4)

**A**


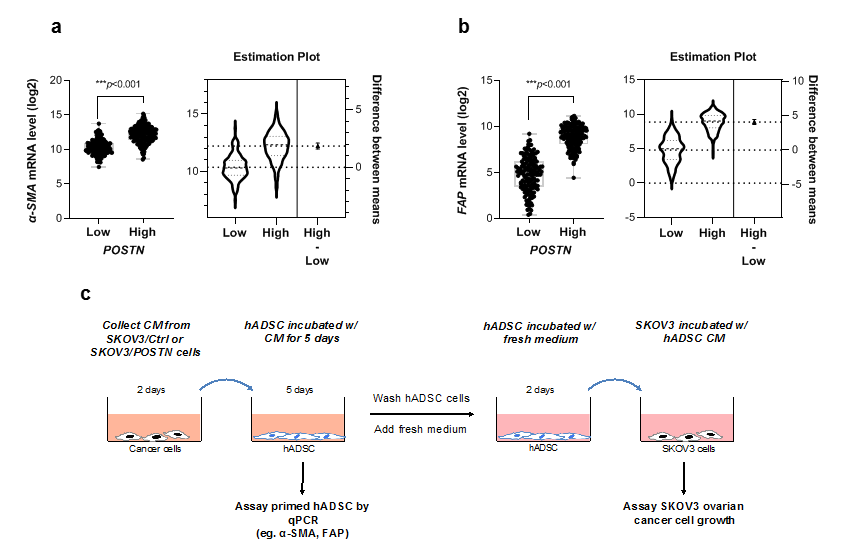


**B**

**C**

**Additional Fig. S5. POSTN expression is associated with increasing** **abundance of cancer associated fibroblasts (CAFs).** The mRNA levels of *POSTN*, *α-SMA* (*ACTA2*) and *FAP* were downloaded from TCGA ovarian cancer (OV) database (n=308). Patients with high and low levels of *POSTN* were divided according to the median expression level of *POSTN*. **A** Box and whiskers plot shows the mRNA levels of *α-SMA* between POSTN-low (n=154) and –high (n=154) ovarian cancer patients (**A**, left). Estimation plot shows the difference between means (**A**, right). **B** Box and whiskers plot shows the mRNA levels of *FAP* between POSTN-low (n=154) and –high (n=154) ovarian cancer patients (**B,** left). Estimation plot shows the difference between means (**B**, right). (Two tailed paired *t*-test, ****P* < 0.001). **C** Schematic diagram of sequential culture of the fibroblast priming assay showing the effect of POSTN primed human adipose derived stem cells (hADSC) on the growth of SKOV3 cells. (related to Fig. 6 G-L)

**Additional Tables**

**Table S1. Reagents used in this study**

| **Reagent** | **Cat. No.** | **Company** |
| --- | --- | --- |
| Recombinant human POSTN protein (rPOSTN) | 3548-F2 | R&D Systems |
|  | | |
| **NF-κB pathway inhibitors** | **Cat. No.** | **Company** |
| [5-(p-fluorophenyl)-2-ureido]thiophene-3-carboxamide (TPCA-1) (Synonyms: IKK-2 Inhibitor IV) | 401481 | Merck/  Calbiochem |
| SN50 peptides (AAVALLPAVLLALLAPVQRKRQKLMP) | 481480 | Merck/  Calbiochem |

**Table S2. siRNA and shRNA clones used in this study**

| **TRC Clone** | **Clone ID** | **Target sequences (5’ to 3’)** |
| --- | --- | --- |
| sh*POSTN* #1 | TRCN0000123055 | CGGTGACAGTATAACAGTAAA |
| sh*POSTN* #2 | TRCN0000123058 | CACTTGTAAGAACTGGTATAA |
|  |  |  |
| si*ITGB5* | Sense | GCGAAAGGAUGCACUGCAUtt |
|  | Anti-sense | AUGCAGUGCAUCCUUUCGCtt |

**Table S3. Antibodies used in this study**

| **Antibody** | **Cat #** | **Company** | **Application** |
| --- | --- | --- | --- |
| POSTN | 19899-1-AP | Proteintech | IHC |
| CD206 | ab64693 | Abcam |  |
| F4/80 | Ab6640 | Abcam |  |
| α-SMA | 19245 | Cell Signaling Technology |  |
|  |  |  |  |
| CD68 | 11-0689 | eBioscience | FACS |
| CD80 | 12-0809 | eBioscience |  |
| CD206 | 17-2069 | eBioscience |  |
|  |  |  |  |
| POSTN | ab14041 | Abcam | WB |
| Actin | SC-56459 | Santa Cruz Biotechnology |  |
| p-Erk1/2  (T202/Y204) | 9101 | Cell Signaling Technology |  |
| Erk1/2 | 9102 | Cell Signaling Technology |  |
| p-p38 MAPK (T180/Y182) | 4511 | Cell Signaling Technology |  |
| p38 MAPK | 8690 | Cell Signaling Technology |  |
| IKKα | 2682 | Cell Signaling Technology |  |
| IKKβ | 2678 | Cell Signaling Technology |  |
| p-NF-κB p65 (S536) | 3033 | Cell Signaling Technology |  |
| NF-κB p65 | SC-109 | Santa Cruz Biotechnology |  |
| ITGB5 | 4708 | Cell Signaling Technology |  |
| Tubulin | SC-23948 | Santa Cruz Biotechnology |  |
| Paxillin | 05-417 | Merck Millipore |  |
| p-Paxillin (Y118) | 2541 (or 69363) | Cell Signaling Technology |  |
| FAK | 05-537 | Merck Millipore |  |
| p-FAK (Y861) | 07-832 | Merck Millipore |  |
| Src | 2109 | Cell Signaling Technology |  |
| p-Src (Y416) | 6943 | Cell Signaling Technology |  |
| TGFβ2 | GTX16335 | GeneTex |  |
| GAPDH | GTX100118 | GeneTex |  |
|  |  |  |  |
| Integrin αVβ3 | MAB1976 | Merck Millipore | Neutralization |
| Integrin αVβ5 | MAB1961 | Merck Millipore |  |

**Table S4. qPCR primers used in this study**

| **Gene** | **Forward sequences (5’-3’)** | **Reverse sequences (5’-3’)** |
| --- | --- | --- |
| *POSTN* | GAACCAAAAATTAAAGTGATTGAAGG | TGACTTTTGTTAGTGTGGGTCCT |
| *actin* | CGGCATCGTCACCAACTG | TCTCAAACATGATCTGGGTCATCT |
| *IL-3* | ttgcctttgctggacttca | ctgttgaatgcctccaggtt |
| *IL-10* | cataaattagaggtctccaaaatcg | aaggggctgggtcagctat |
| *IL-12* | cactcccaaaacctgctgag | caatctcttcagaagtgcaagg |
| *IL-16* | ggaagggctccctacacg | cacccagctgcaagatttc |
| *MIP-1β* | ctctccagcgctctcagc | accacaaagttgcgaggaag |
| *MCP-1* | agtctctgccgcccttct | gtgactggggcattgattg |
| *MCSF* | agtccgagggatcctcca | cgctctctgaggctcttgat |
| *TNFα* | agcccatgttgtagcaaacc | tctcagctccacgccatt |
| *RANTES* | tgcccacatcaaggagtattt | ctttcgggtgacaaagacg |
| *CCL2* | cagccagatgcaatcaatgcc | tggaatcctgaacccacttc |
| *CCL10* | gtggcattcaaggagtacctc | Tgatggccttcgattctggatt |
| *CCL11* | gacgctgtctttgcataggc | ggatttaggcatcgttgtccttt |
| *CCL17* | atggccccactgaagatgctt | tgaacaccaacggtggagg |
| *CCL18* | gggggctggtttcagaata | ctccttgtcctcgtctgcac |
| *CCL22* | ggaggcaaagagtagggtgtaat | tcagccagaaaggcatagata |
| *MMP-2* | ccccaaaacggacaaagag | cttcagcacaaacaggttgc |
| *MMP-9* | gaaccaatctcaccgacagg | gccacccgagtgtaaccata |
| *IL-1α* | ggttgagtttaagccaatcca | tgctgacctaggcttgatga |
| *IL-1β* | tacctgtcctgcgtgttgaa | tctttgggtaatttttgggatct |
| *α-SMA* | CTATGCCTCTGGACGCACAACT | CAGATCCAGACGCATGATGGCA |
| *FAP* | GGAAGTGCCTGTTCCAGCAATG | TGTCTGCCAGTCTTCCCTGAAG |

**Table S5. Summary table of TissueScan cohort (#HORT102, OriGene)**

| **Clinical variable** | **All** | **Normal** | **Subtypes** | | | |
| --- | --- | --- | --- | --- | --- | --- |
|  |  |  | **Serous** | **Clear cell** | **Endometrioid** | **Mucinous** |
| Number of cases | 48 | 8 (16.67%) | 33 (68.75%) | 1 (2.08%) | 5 (10.42%) | 1 (2.08%) |
| **Age** |  | | | | | |
| > 40 | 45 (93.75%) | 5 (62.50%) | 33 (100%) | 1 (100%) | 5 (100%) | 1 (100%) |
| ≤ 40 | 3 (6.25%) | 3 (37.50%) | --- | --- | --- | --- |
| **Stage** |  | | | | | |
| Stage I | 8 (20.00%) | --- | 4 (12.12%) | 1 (100%) | 3 (60.00%) | 0 |
| Stage II | 9 (22.50%) | --- | 8 (24.24%) | 0 | 1 (20.00%) | 0 |
| Stage III | 17 (42.50%) | --- | 15 (45.45%) | 0 | 1 (20.00%) | 1 (100%) |
| Stage IV | 6 (15.00%) | --- | 6 (18.18%) | 0 | 0 | 0 |

**Table S6. Summary table of ovarian cancer patients from CMUH (IRB#: CMUH 107-REC1-095)**

| **Clinical variable** | **All** | **Subtypes** | | | |
| --- | --- | --- | --- | --- | --- |
|  |  | **Serous** | **Clear cell** | **Endometrioid** | **Mucinous** |
| Number of cases | 65 | 64 (98.46%) | 0 | 0 | 1 (1.54%) |
| **Age** |  | | | | |
| > 40 | 61 (93.85%) | 60 (93.75%) | 0 | 0 | 1 (100 %) |
| ≤ 40 | 4 (6.15%) | 4 (6.25%) | 0 | 0 | 0 |
| **Stage** |  | | | | |
| Stage I | 7 (10.77%) | 6 (9.38%) | 0 | 0 | 1 (100%) |
| Stage II | 8 (12.30%) | 8 (12.50%) | 0 | 0 | 0 |
| Stage III | 41 (63.08%) | 41 (64.06%) | 0 | 0 | 0 |
| Stage IV | 9 (13.85%) | 9 (14.06%) | 0 | 0 | 0 |
